# Supplementary material for: How are estimated cellular turnover rates influenced by the dynamics of a source population?
Source: PLoS Comput Biol. 2025 May 12;21(5):e1013052. doi: 10.1371/journal.pcbi.1013052 (PMC12129353; doi:10.1371/journal.pcbi.1013052)
Supplement: S1 Text — The loss rate of the POI was chosen to be 20 times higher than that of the precursors (d1=0.5, d2=10). Note that the slopes are calculated as the label (gain or) loss rate multiplied by the fraction of (un)labelled cells, for example, d*(1+∈)l2. The division rate of the POI, p2, was either set to 5 (in (a) and (c)) or 0 (in (b) and (d)). Fig B: Calculated gain rates are a good approximation of the true labelling curves if precursors are faster than the POI. The turnover of deuterium in body water has a 10-day timescale in these simulations. See Fig 1 in the main text for other details. Note that the body water deuterium concentration, D(t), has been plotted on a different axis (on the right) for easier comparison to Fig 1 in the main text. Fig C: Calculated gain rates are a good approximation of the true labelling curves if precursors are slower than the POI. The turnover of deuterium in body water has a 10-day timescale in these simulations. See Fig 2 in the main text for other details. Note that the body water deuterium concentration, D(t), has been plotted on a different axis (on the right) for easier comparison to Fig 2 in the main text. Fig D: The estimated labelling rates if the POI is very short-lived. The turnover of deuterium in body water has a 10-day timescale in these simulations. See Fig A in S1 Text above for other details. Note that the body water deuterium concentration, D(t), has been plotted on a different axis (on the right) for easier comparison to Fig A in S1 Text above. Fig E: The best fits of the phenomenological model (Equation 8 in the main text) to the labelling curve of the POIs shown in Fig 1. The black circles show the data, and the red trajectory shows the best fit. Fig F: The best fits of the phenomenological model (Equation 8 in the main text) to the labelling curve of the POIs shown in Fig 2. The black circles show the data, and the red trajectory shows the best fit. Fig G: Examples of the best fits used to generate Fig 3. The top ro [file pcbi.1013052.s001.pdf]

Supplementary information

Table A

d_1	d_2	p_2	k	$p^*(0)$	$p^*(1)$	$d^*(1+\epsilon)$
0.5	10	5	1	5	0.68	6.33
			2	7.5	0.80	8.43
		0	1	0	0.46	-1
			2	5	0.68	6.33

Table A: The true and calculated rates corresponding to the simulations shown in Fig A. The rates (expressed as stu) are scaled with respect to the labelling period.

<u>The initial gain and loss rates are well-described even when the POI is very short-lived</u>

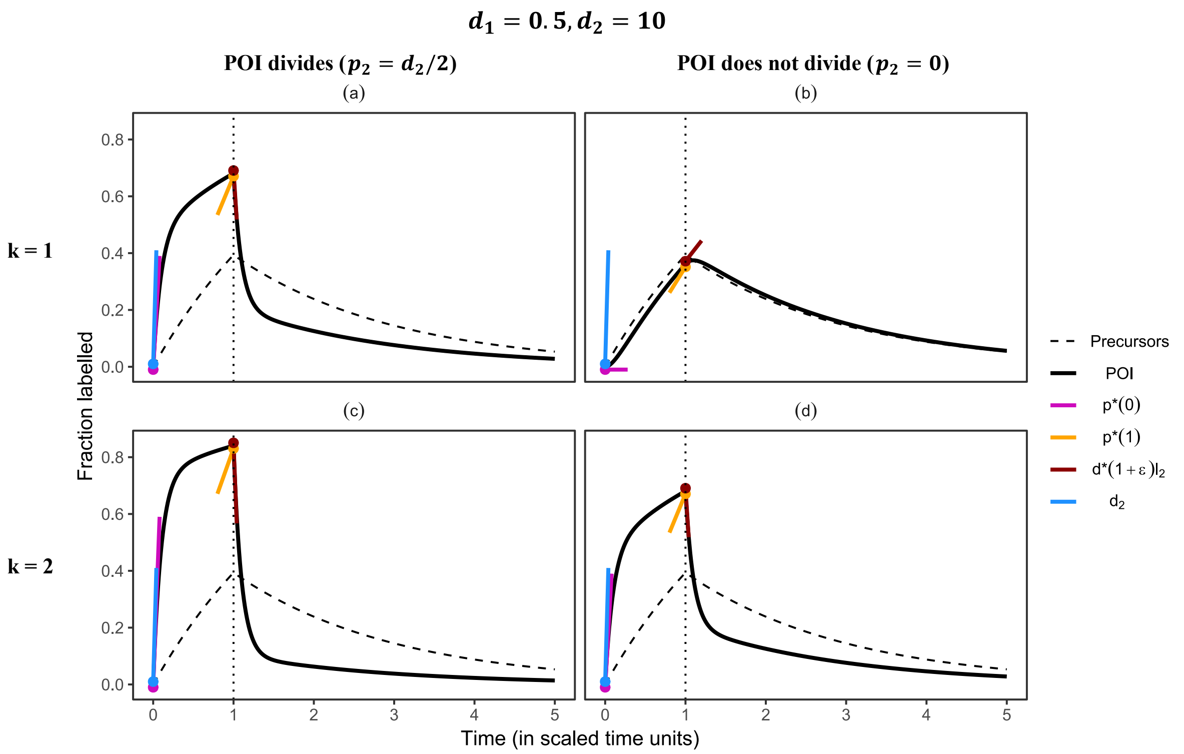

Fig A: The estimated labelling rates when the POI is very short-lived. The loss rate of the POI was chosen to be 20 times higher than that of the precursors ($d_1 = 0.5$, $d_2 = 10$). Note that the slopes are calculated as the label (gain or) loss rate multiplied by the fraction of (un)labelled cells, for example, $d^*(1+\epsilon)l_2$. The division rate of the POI, p_2, was either set to 5 (in (a) and (c)) or 0 (in (b) and (d)).

The calculated initial gain and loss rates provide decent approximations of the observed initial gain and loss rates (in most cases) even if $D(t)$ is not a square pulse

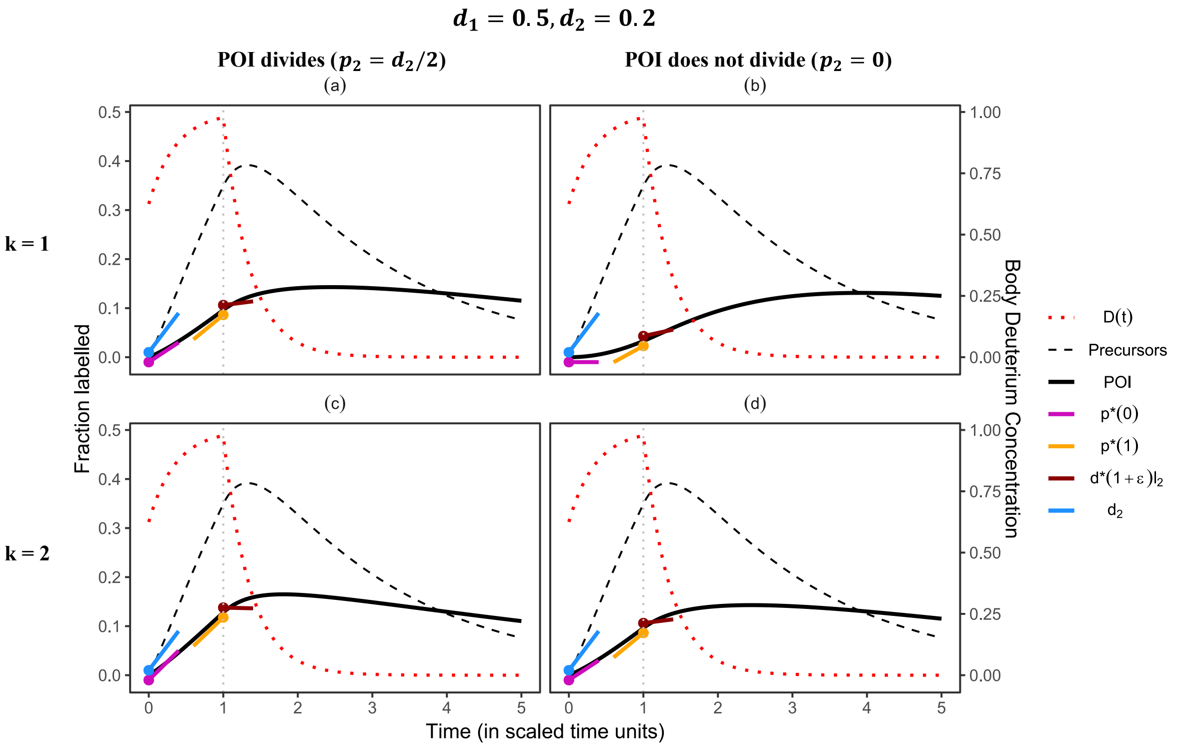

Fig B: Calculated gain rates are a good approximation of the true labelling curves if the precursors are faster than the POI. The turnover of deuterium in body water has a 10-day timescale in these simulations. See Fig 1 in the main text for other details. Note that the body water deuterium concentration, $D(t)$, has been plotted on a different axis (on the right) for easier comparison to Fig 1 in the main text.

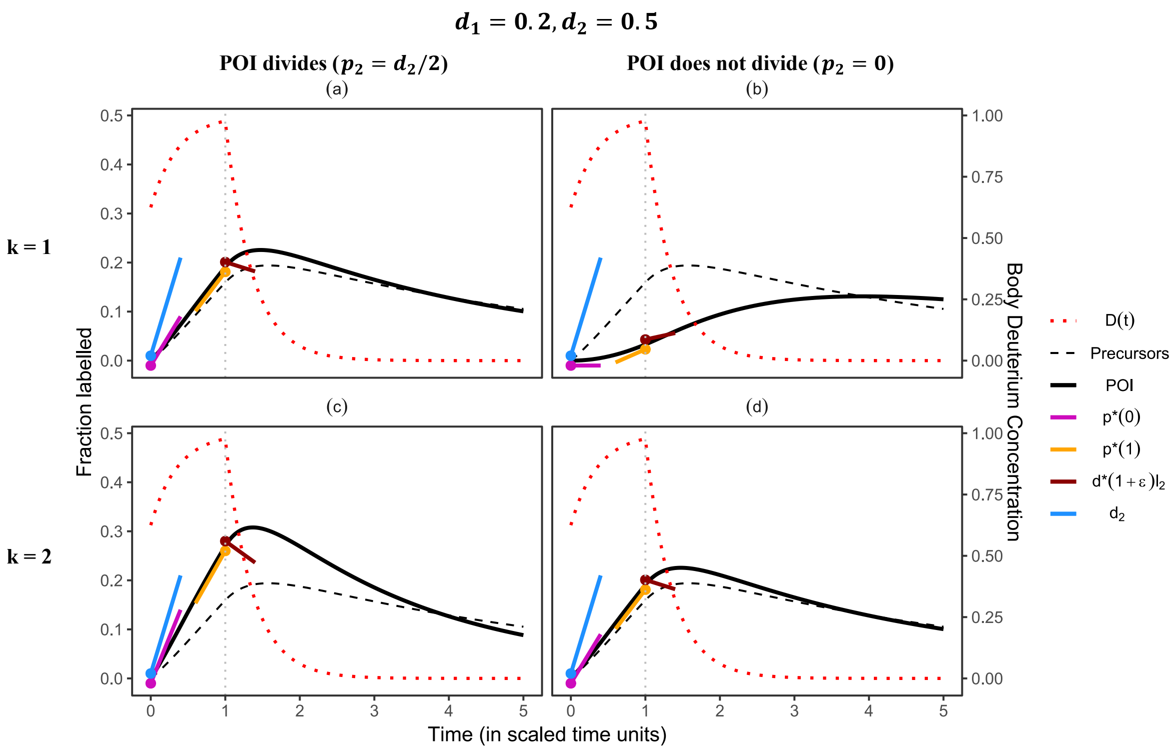

Fig C: Calculated gain rates are a good approximation of the true labelling curves if the precursors are slower than the POI. The turnover of deuterium in body water has a 10-day timescale in these simulations. See Fig 2 in the main text for other details. Note that the body water deuterium concentration, $D(t)$, has been plotted on a different axis (on the right) for easier comparison to Fig 2 in the main text.

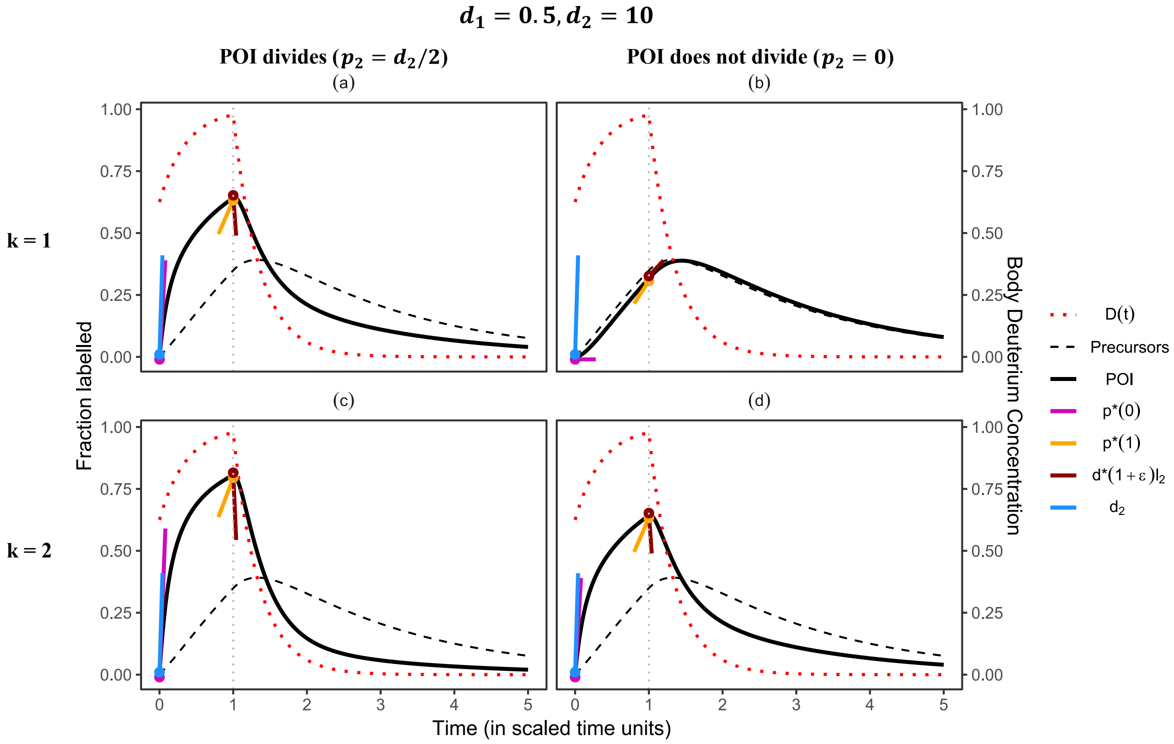

Fig D: The estimated labelling rates if the POI is very short-lived. The turnover of deuterium in body water has a 10-day timescale in these simulations. See Fig A above for other details. Note that the body water deuterium concentration, $D(t)$, has been plotted on a different axis (on the right) for easier comparison to Fig A above.

<u>The best description of the labelling in the POI by the phenomenological p^*d^* model</u>

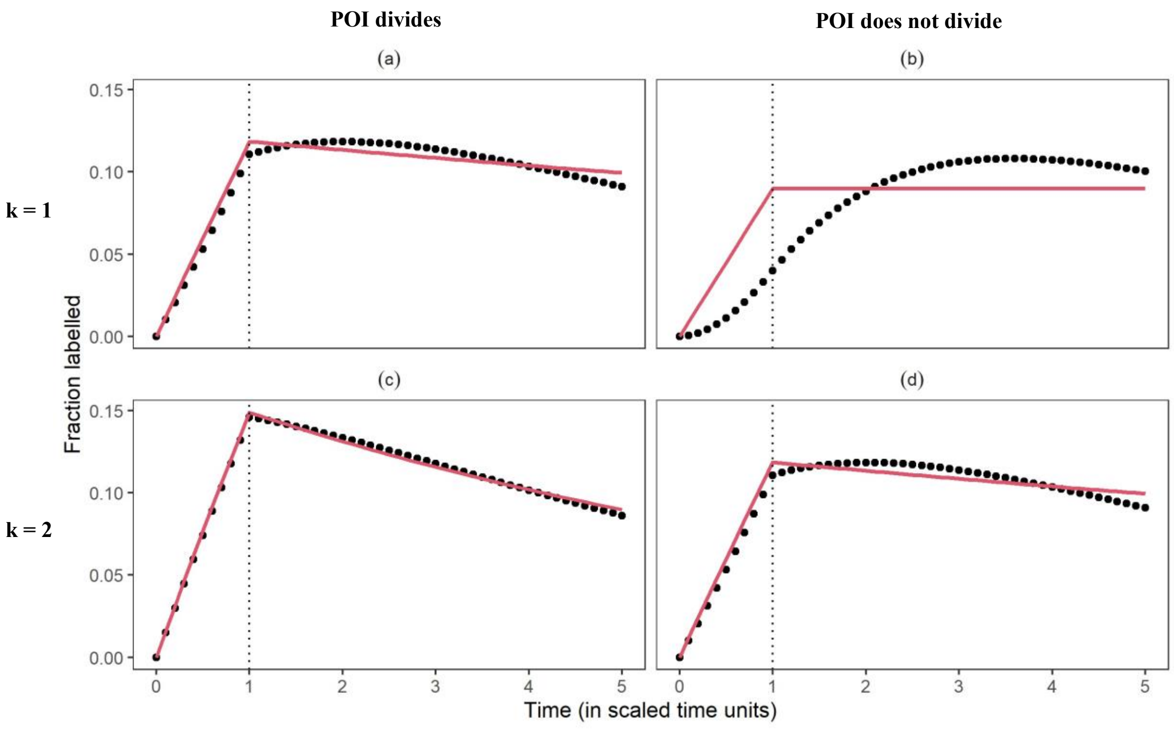

Fig E: The best fits of the phenomenological model (equation 8 in the main text) to the labelling curve of the POI shown in Fig 1. The black circles show the data, and the red trajectories show the best fits. The in silico data were saved every 0.1 time unit, such that each plot shows 50 data points.

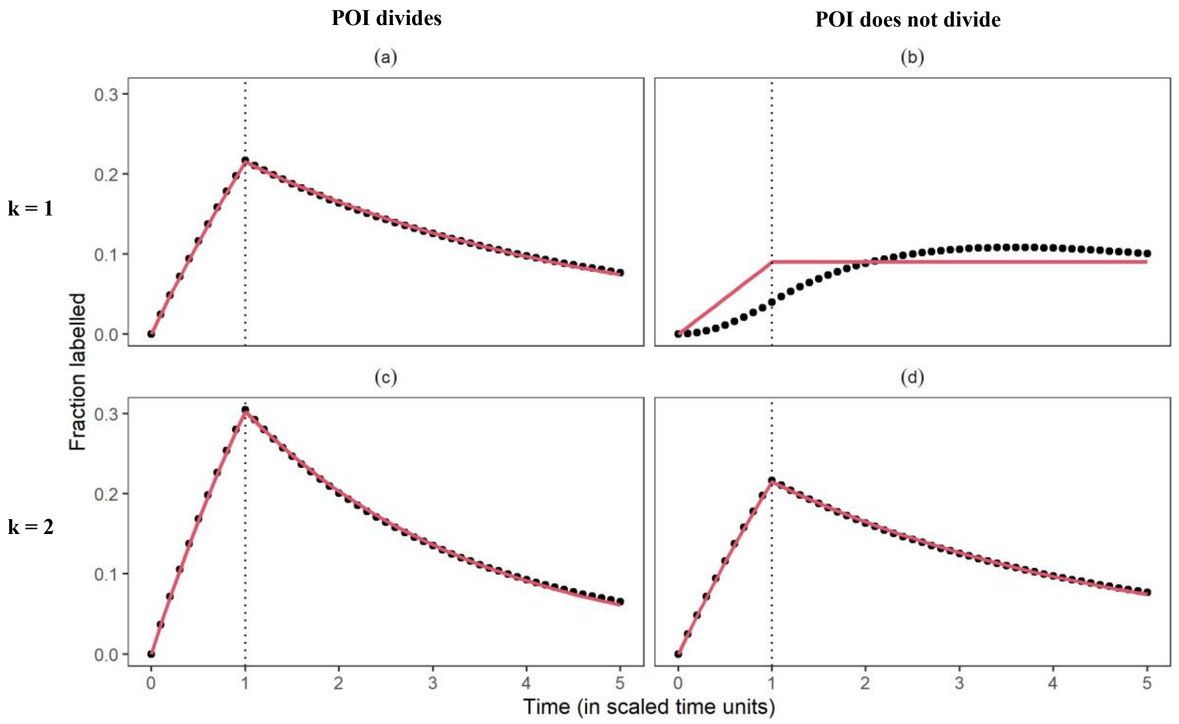

Fig F: The best fits of the phenomenological model (equation 8 in the main text) to the labelling curve of the POIs shown in Fig 2. The black circles show the data, and the red trajectory shows the best fit. The in silico data were saved every 0.1 time unit, such that each plot shows 50 data points.

<u>Representative fits of the implicit source model (equation 8) for the $k = 1$ case</u>

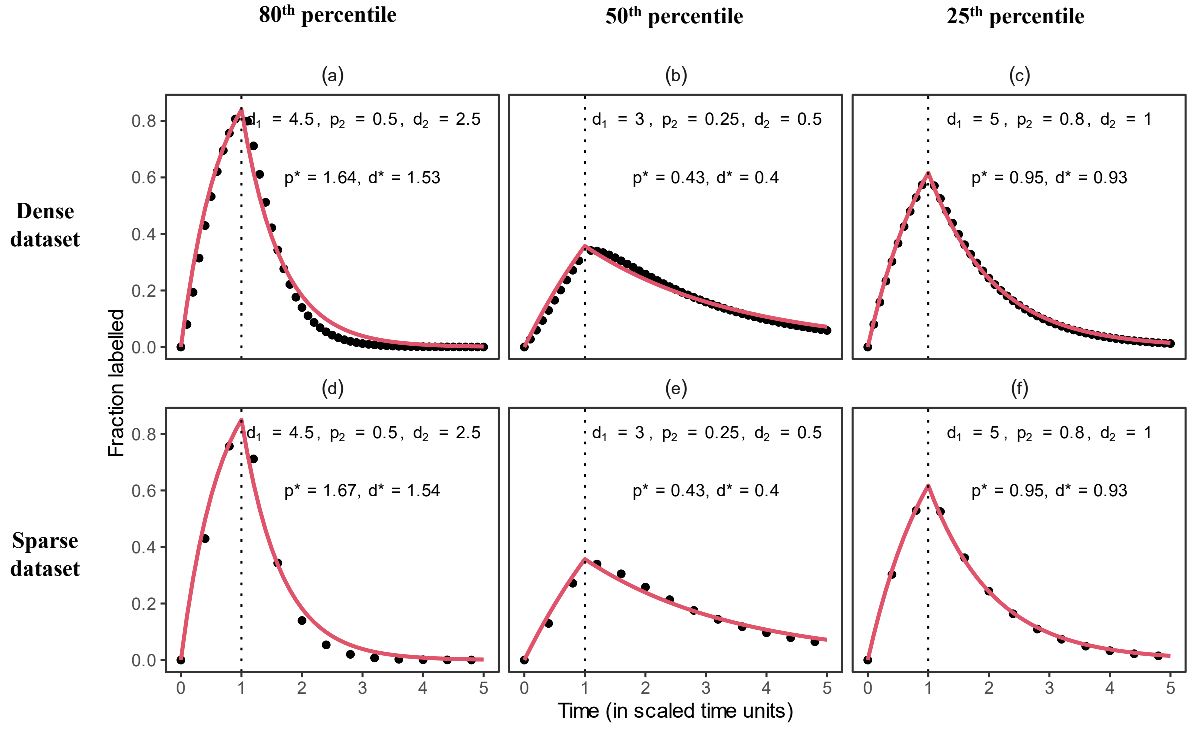

Fig G: Examples of the best fits used to generate Fig 3. The top row shows fits to dense data and the bottom row shows fits to sparse data. Note that the fits and the estimates hardly change if the data is made sparser. The synthetic data were saved every 0.1 time unit in the top row and every 0.4 time unit in the bottom row, such that the plots in the top row show 50 data points and the plots in the bottom row show 13 data points.

Correlation plots and representative fits of the implicit source model (equation 8) for the $k = 1$ case based on noisy data

True experimental data is noisy. Here, we test whether the relationships between the true parameters and the estimated parameters are significantly different if the artificial dataset is ‘noisy'.

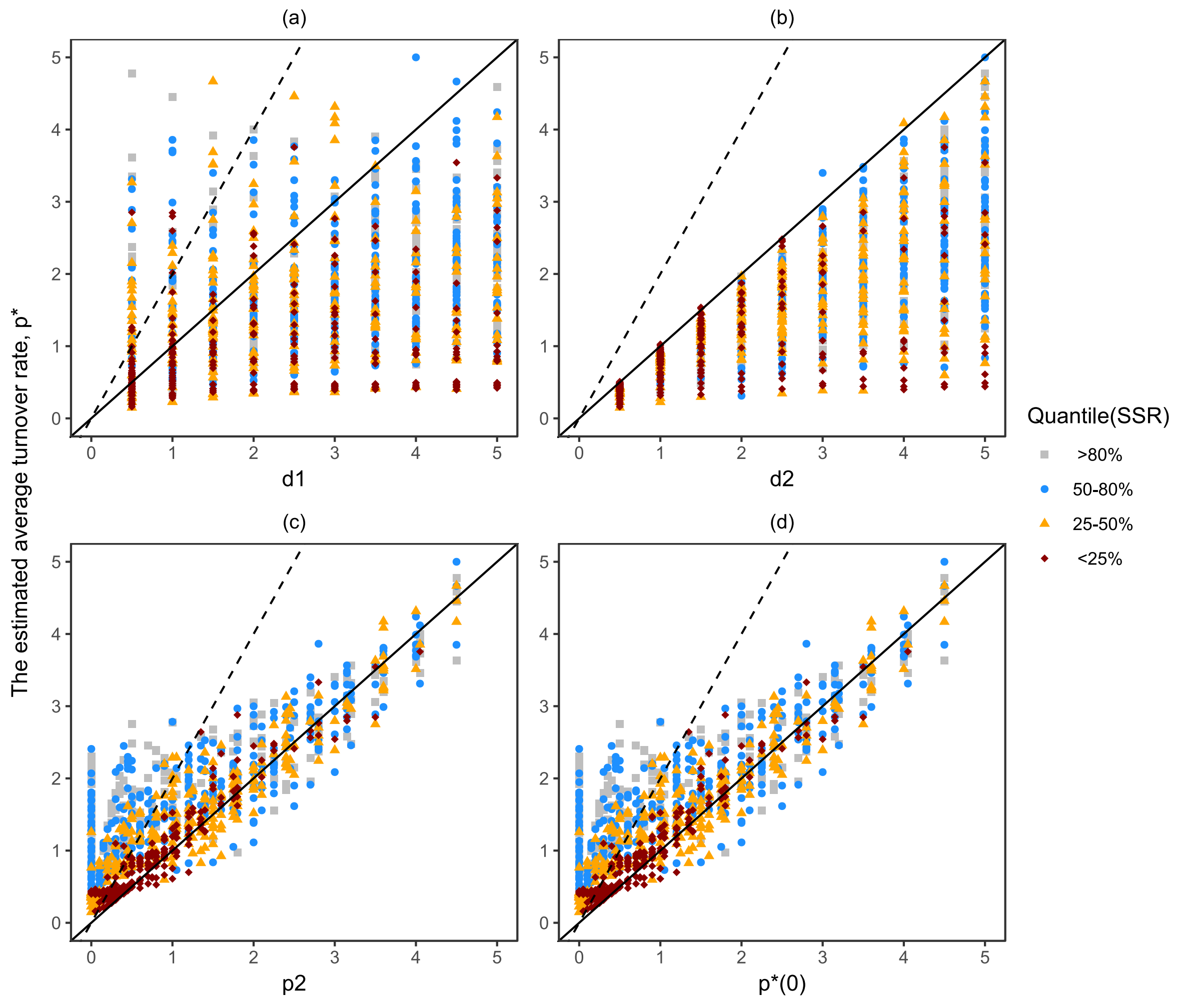

Fig H: Recreation of Fig 3 of the main text with ‘noisy’ data points. The same as Fig 3 in the main text, except that each data point in the artificially generated dataset is replaced by two ‘noisy’ data points. The two noisy data points are drawn from a normal distribution with

a mean that equals the original data point and a 20% standard deviation. The solid and dashed lines represent slopes of 1 and 2, respectively.

The conclusions drawn from the noisy dataset are the same as those drawn from the 'clean' dataset. However, the noisy dataset exaggerates the quantitative behaviour, i.e., the difference between the estimated p^* and the true value of p_2 is larger due to the noisy dataset (Fig Hd).

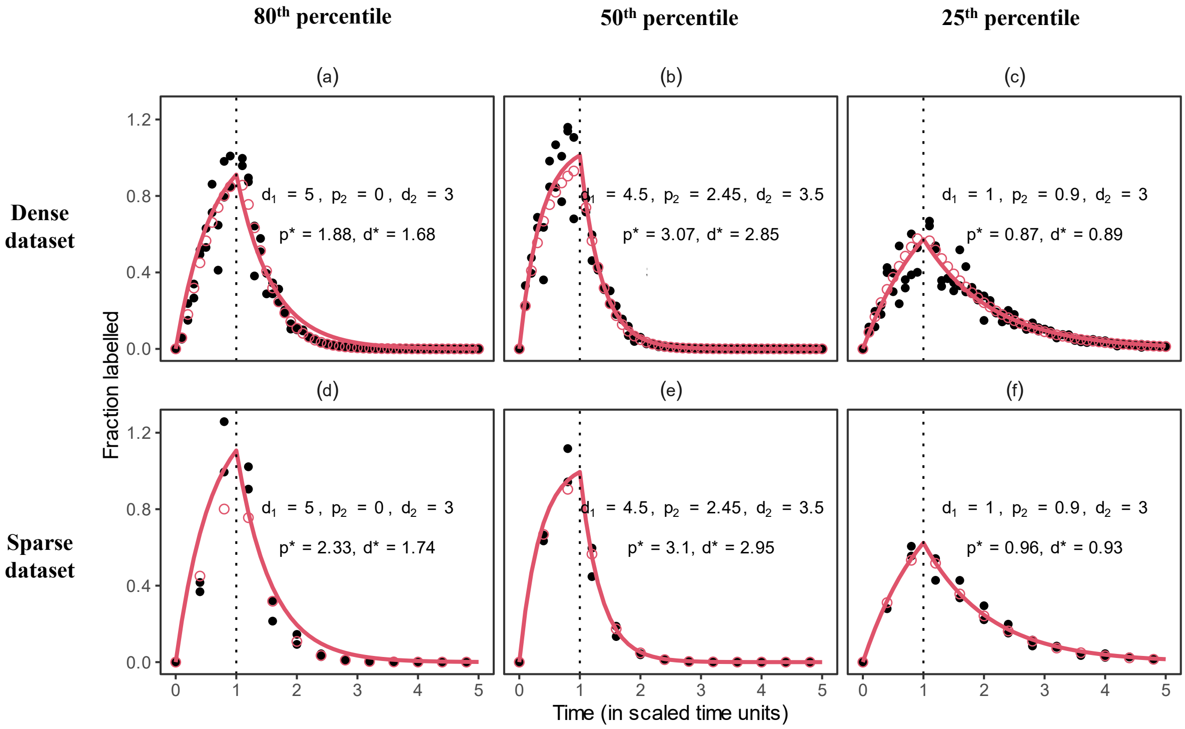

Fig I: Examples of the best fits used to generate Fig H. The top row shows fits to dense data and the bottom row shows fits to sparse data. Note that the fits and the estimates hardly change if the data is made more sparse. The open red circles show the data points without noise added to them, while the black bullet points are the data points after noise was introduced. There are two black bullet points corresponding to each open red circle. The model was fitted to the black bullet points. The in silico data were saved every 0.1 time unit in the top row and every 0.4 time unit in the bottom row, such that the plots in the top row show 50 data points and the plots in the bottom row show 13 data points.

Representative fits of the implicit source model (equation 8) for the $k = 2$ case

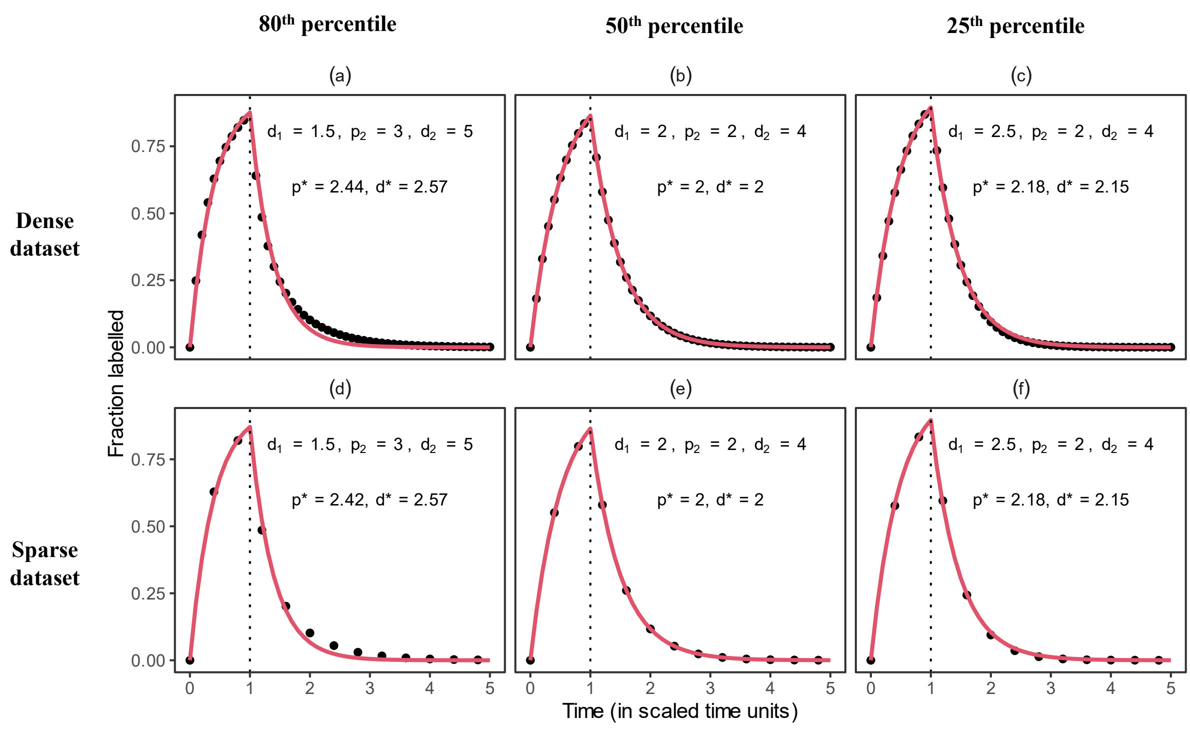

Fig J: Examples of the best fits used to generate Fig 4. The top row shows fits to dense data and the bottom row shows fits to sparse data. Note that the fits and the estimates hardly change if the data is made more sparse. The in silico data were saved every 0.1 time unit in the top row and every 0.4 time unit in the bottom row, such that the plots in the top row show 50 data points and the plots in the bottom row show 13 data points.

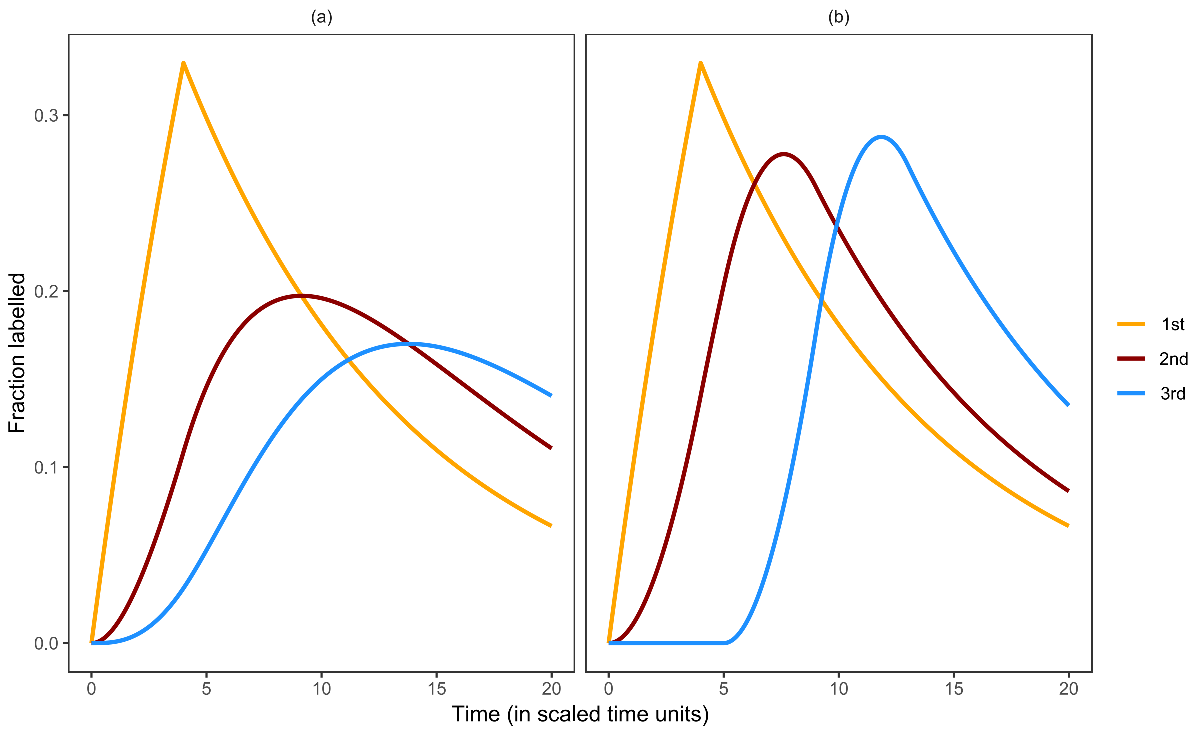

Fig K: Non-dividing populations with the same differentiation properties show different behaviours if they have pre-programmed cellular processes. A 3-population system is shown where cells of either all populations ((a), equation C) or only the first population ((b), equation D) have random cellular processes. The parameters of the models are: $d_1 = 0.1$, $\frac{1}{d_2} = \Delta_2 = 5$, $\frac{1}{d_3} = \Delta_3 = 4$. The values are scaled with respect to the labelling period.

Model fit of the two sub-population kinetic heterogeneity model and the ES model to the $CD57^+$ $CD4^+$ memory T-cell data for $k = 2$

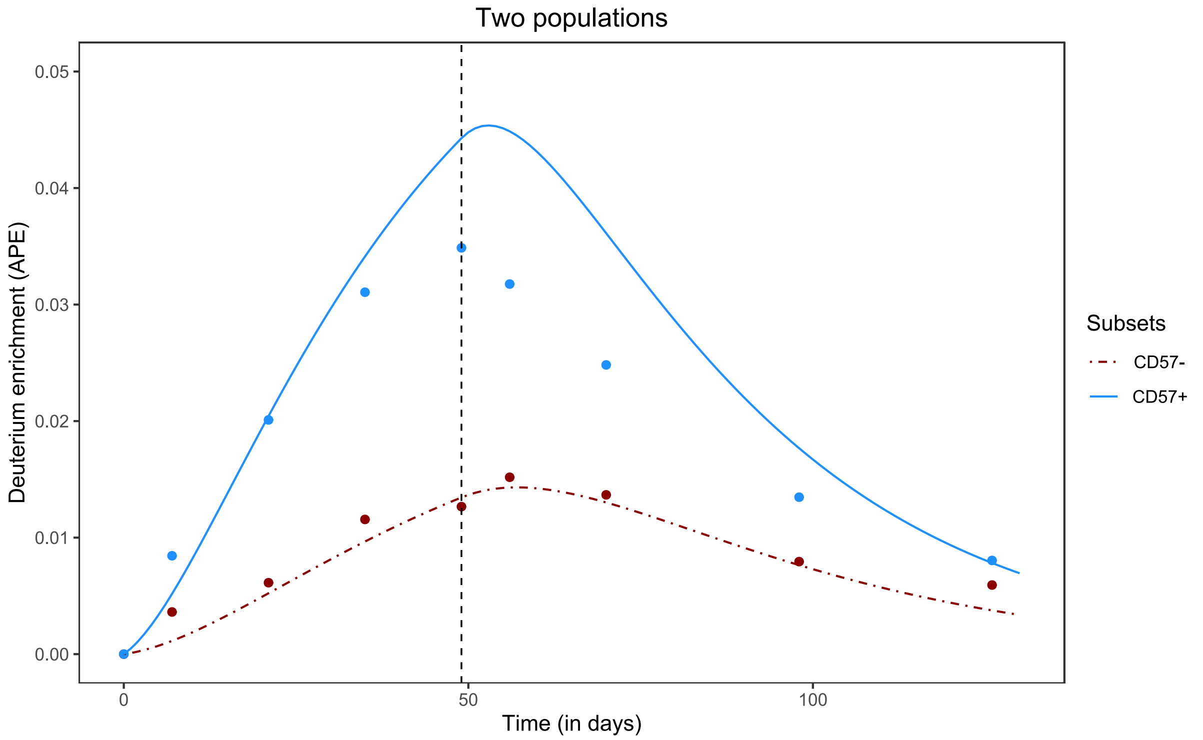

Fig L: The best fit gives a poor description of the labelling data of CD4+ CD57+ memory T cells for $k = 2$. The lines depict the best fit of the two sub-population kinetic heterogeneity and the one population ES model to the deuterium labelling data of $CD57^-$ $CD4^+$ and $CD57^+CD4^+$ memory T cells of individual DW02 from Ahmed et al. (2020) [1]. The labelling data of the cell populations and the data of the body water deuterium concentration were digitized from the original article for re-analysis. See the caption of Fig 6 in the main text for additional details.

Text A

The ES model describes both non-stem-like and stem-like populations

The ES model makes only the most fundamental assumptions (of a possible source, of possible division in the population, and of loss from the population), that should be true for any population and, thus, is very general. With a few substitutions, we can also express the system of equations (equations 2a-d) in the classical form of stem cells [2], i.e.,

$$\frac{dN_1}{dt} = \sigma + (p_1 - d_1)N_1$$

$$= \sigma - (1-\alpha)d_1N_1 - \alpha d_1 N_1 - p_1N_1 + 2p_1N_1$$

$$= \sigma - (1-\alpha)d_1N_1 + (2\beta - 1)\delta N_1 \quad \text{(A1)}$$

$$\frac{dN_2}{dt} = 2\alpha d_1 N_1 + (p_2 - d_2)N_2$$

$$= 2(1-\beta)\delta N_1 + (p_2 - d_2)N_2 \quad \text{(A2)}$$

where $\delta = p_1 + \alpha d_1$ is the total division rate of the precursor population, and $\beta = \frac{p_1}{\delta} < 1$ is the fraction of the daughter cells that replenish the precursor population. The fraction $(1-\beta)$ of daughter cells differentiate into the next population, N_2. Therefore, the ES model is general enough to describe both non-stem-like and stem-like populations.

Text B

The label gain rate can maximally be the turnover rate of the POI

The normalized label gain rate, $q(t) = \frac{p^*(t)}{d_2}$, is (from equation 12a):

$$q(t) = \frac{ab}{(1-b)}e^{-d_1t} + \left(1 - \frac{a}{(1-b)}\right)e^{-d_2t} \quad \text{(B1)}$$

where, $a = \frac{(1-\alpha_2)}{k}$, $b = \frac{d_1}{d_2}$ and $\alpha_2 = \frac{p_2}{d_2}$. To find the critical time, t_c, where the curve $q(t)$ is either at its minimum or at its maximum, we can equate the first derivative of $q(t)$ to zero, i.e., $\frac{dq(t)}{dt}|_{t=t_c} = 0$, which leads to the following condition:

$$\left(1 - \frac{a}{(1-b)}\right)d_2e^{-d_2t_c} = -\frac{ab}{(1-b)}d_1e^{-d_1t_c} \quad \text{(B2)}$$

The curve $q(t)$ is at its maximum at the timepoint t_c as the second derivative of $q(t)$ is negative, i.e., $\frac{d^2q(t)}{dt^2}|_{t=t_c} < 0$, when the above condition is satisfied (equation B2).

To find the maximum of the curve $q(t)$, observe that (from equations B1-2):

$$q(t_c) = -\left(1 - \frac{a}{(1-b)}\right)\frac{1}{b}e^{-d_2 t_c} + \left(1 - \frac{a}{(1-b)}\right)e^{-d_2 t_c} \quad \text{(B3)}$$

$$= \left(1 - \frac{1}{b}\right)\left(1 - \frac{a}{(1-b)}\right)e^{-d_2 t_c} \quad \text{(B4)}$$

$$= \left(1 - \frac{(1-a)}{b}\right)e^{-d_2 t_c} \quad \text{(B5)}$$

As we reason in the main text, $q(t_c) < 1$ as $\left(1 - \frac{(1-a)}{b}\right) < 1$ when $a < 1$, which is always true. Thus, the maximum of the curve $q(t)$ is less than 1, and $p^*(t) < d_2$.

Text C

Labelling curves for chains of cell populations with non-random (or fixed) cellular processes

In the main text, we have focused on scenarios where cells are lost or are produced randomly (i.e., the times to death or division are exponentially distributed and are independent of the cell's age). However, several cellular processes may require a minimum length of time. If the cells of the POI were to have an 'internal clock' programming their departure time, i.e., if cells would require a fixed amount of time to mature or die, then the peak of labelling achieved in the POI can be higher than that in the precursors (Fig K).

Consider the kinetics of this 3-population system where the population is governed by fixed cellular processes:

$$\frac{dN_1(t)}{dt} = \sigma - d_1 N_1(t) \quad \text{(C1)}$$

$$\frac{dN_2(t)}{dt} = d_1 N_1(t) - d_1 N_1(t - \Delta_2) \quad \text{(C2)}$$

$$\frac{dN_3(t)}{dt} = d_1 N_1(t - \Delta_2) - d_1 N_1(t - \Delta_2 - \Delta_3) \quad \text{(C3)}$$

with, $$\overline{N_1} = 1, \overline{N_2} = d_1 \overline{N_1} \Delta_2, \text{ and } \overline{N_3} = d_1 \overline{N_1} \Delta_3 \quad \text{(C4)}$$

The residence times in N_1, N_2 and N_3 are $1/d_1$, Δ_2 and Δ_3, respectively. The dynamics of the labelled fraction are defined as

$$\frac{dl_1}{dt} = d_1(D(t) - l_1) \tag{C5}$$

$$\frac{dl_2}{dt} = \frac{l_1(t) - l_1(t - \Delta_2)}{\Delta_2} \tag{C6}$$

$$\frac{dl_3}{dt} = \frac{l_1(t - \Delta_2) - l_1(t - \Delta_2 - \Delta_3)}{\Delta_3} \tag{C7}$$

where

$$l_1(t - \Delta) = 0, \text{ if } t < \Delta \tag{C8}$$

Here a population, N_1, governed by random cellular processes (i.e., an exponentially distributed loss rate, d_1) differentiates into a population, N_2, which with fixed maturation time (Δ_2) differentiates into a successive stage, N_3, which also has a fixed maturation time (Δ_3).

As the cells only mature in the 2nd and 3rd populations (the non-random populations) and do not divide, the gain of label in these populations is solely due to differentiation of the 1st population (with random cellular processes). The 2nd population gathers label as soon as labelling starts, as there is no fixed time in the 1st population before which a cell can mature. However, the 3rd population gains label only after the labelled cells in the 2nd population have matured, i.e., after Δ_2 units of time.

The dynamics of an analogous system where the three populations have random cellular processes is:

$$\frac{dN_1(t)}{dt} = \sigma - d_1 N_1(t)$$

$$\frac{dN_2(t)}{dt} = d_1 N_1(t) - d_2 N_2(t)$$

$$\frac{dN_3(t)}{dt} = d_2 N_2(t) - d_3 N_3(t)$$

$$\overline{N_1} = \frac{\sigma}{d_1};\ \overline{N_2} = \frac{d_1}{d_2}\overline{N_1};\ \overline{N_3} = \frac{d_2}{d_3}\overline{N_2} \tag{D}$$

$$\frac{dl_1(t)}{dt} = d_1(D(t) - l_1(t))$$

$$\frac{dl_2(t)}{dt} = d_2(l_1(t) - l_2(t))$$

$$\frac{dl_3(t)}{dt} = d_3(l_2(t) - l_3(t))$$

In a 3-population system that only has populations with random cellular processes (Fig K), the successive population (2nd and 3rd) attain their peak where their labelling curves intersect with their immediate precursors, and therefore, the peak of a successive population cannot be higher than its immediate precursor. This property, however, does not hold in a system that has populations on a conveyor belt (Fig K) as the peak of the 3rd population can be higher than that of the 2nd. Of note, the peak of the 2nd and 3rd population cannot be higher than that of the 1st population (i.e., the source of their labelling). In the simulation presented here, as the maturation time in the 3rd population is shorter than that of the 2nd, the 3rd population has a higher fraction that is labelled at its peak (even though the total amount of labelled cells going through the is the same). Since these curves are different, one would obtain different parameter estimates if such labelling data were fitted with equations C and D. Therefore, it is also important to know whether the measured populations undergo non-random cellular processes.

In the case of non-random cellular processes, the 3rd population has a delayed gain of label (therefore, no proliferation) and peaks well after the stop of the labelling period (so, the source, i.e., the 2nd population, is the major contributor of label). Moreover, its peak is higher than that of its precursor population, which is not possible for populations with random cellular processes. In such a case, one should choose equation C to explain the data. Finally, it is also clear from the marked delay in the labelling of the 3rd population, that the 2nd population is a 'waiting box'.

Text D

Equations of the models used to fit the deuterium labelling curves of the $CD4^+$ $CD57^-$ and $CD4^+$ $CD57^+$ cell populations shown in Figs 6 and L

$$\frac{dl_1(t)}{dt} = d_1(D(t) - l_1(t)) \tag{E1}$$

$$\frac{dl_2(t)}{dt} = d_2(D(t) - l_2(t)) \tag{E2}$$

$$\frac{dl_3(t)}{dt} = \alpha \frac{dl_1(t)}{dt} + (1-\alpha)\frac{dl_2(t)}{dt} \tag{E3}$$

$$\frac{dl_4(t)}{dt} = \frac{(d_4 - p_4)}{k_4}(l_3(t) + k_4 - 1) + p_4 - d_4 l_4(t) \tag{E4}$$

$$\frac{dl_5(t)}{dt} = \frac{(d_5 - p_5)}{k_5}(l_4(t) + k_5 - 1) + p_5 - d_5 l_5(t) \tag{E5}$$

where, $k_4 = k_5$. Equations E1-E3 denote a two sub-population kinetic heterogeneity model and equations E4 and E5 are ES models. Equations E1-E3 were used to fit the data in panel

(a) Single population, equations E1-E4 were used to fit the data in panel (b) Two populations, and equations E1-E5 were used to fit the data in panel (c) Three populations.

References

1. Ahmed R *et al.* 2020 CD57+ Memory T Cells Proliferate In Vivo. *Cell Rep.* **33**. (doi:10.1016/J.CELREP.2020.108501)

2. Lander AD, Gokoffski KK, Wan FYM, Nie Q, Calof AL. 2009 Cell Lineages and the Logic of Proliferative Control. *PLOS Biol.* **7**, e1000015. (doi:10.1371/JOURNAL.PBIO.1000015)
