# Supplementary material for: How are estimated cellular turnover rates influenced by the dynamics of a source population?
Source: PLoS Comput Biol. 2025 May 12;21(5):e1013052. doi: 10.1371/journal.pcbi.1013052 (PMC12129353; doi:10.1371/journal.pcbi.1013052)
Supplement: Attachment — Submitted filename: ChainRebuttal.pdf [file pcbi.1013052.s003.pdf]

Response in red; Referenced line numbers are accurate for the final manuscript without any track changes.

Your manuscript has been carefully reviewed by three independent scientists, who made some pertinent comments and suggestions. Overall, the feedback is positive, but addressing some of the issues is very important. In particular, please emphasize novelty of work versus your previous work and the unpublished Yan et al paper mentioned by multiple reviewers.

We have addressed all the comments from the reviewers below. In addition to the reviewer's comments, we found that we had mistakenly repeated Figure 3 instead of Figure 4 in the submitted manuscript and have now corrected that error in the revised version.

Reviewer's Responses to Questions

Comments to the Authors:

Please note here if the review is uploaded as an attachment.

Reviewer #1: Positive feedback. The paper is well written for the most parts and I could follow mathematical arguments of the authors (although I must admit I did not repeat their derivations). Figures are ok readable (captions need to be improved) and combination of analytical results with simulations is nice. The overall topic is somewhat reasonable (although for those of us studying lymphocyte turnover conclusions are pretty obvious). An example of how one could experimentally discriminate between alternative models (peak of labeling) is a nice example of good thinking, and I encourage authors to do more of that.

We would like to thank the reviewer for his/her supportive comments. We have addressed his/her concerns and provide details of the same below.

Major comments

Specifics of modeling the source/precursors. The authors assumed that there is only one set of precursors before POI. This is probably biologically unrealistic for immune cells (e.g., lymphocytes, neutrophils, etc) since there are many steps where cells move from less differentiated state to more differentiated state. How incorporating n precursor populations, rather than 1, changes the modeling predictions? Please explore.

We mentioned briefly in the submitted version that the labelling dynamics in the POI is not affected by any precursor population apart from its immediate precursor population (see line numbers 464-468 in the unrevised manuscript). We agree with the reviewer that this is an

important point to address and have given it more emphasis in the revised manuscript (see line numbers 141-149 (discussion around equation 2h) in the revised manuscript).

Impact of deuterium dynamics on results. All results are generated assuming that labeling is described by the step function. For example, the prediction that one could discriminate between alternative models is due to peak of labeling curves is interesting but I am not sure if it would survive given complexity of deuterium accumulation over time in the system/blood. Please investigate if this complexity would negate ability to discriminate between alternatives.

The reviewer is correct. There is definitely an effect of the body deuterium concentration on our results. The results shown in the manuscript assume a step function, which is a good approximation of the build-up and loss of deuterium in body glucose. The washout rate of deuterium in body water, although slower than that in body glucose (which has a 30-minutes timescale in humans), is still quite fast (i.e., a 4-day timescale in mice and 10-day timescale in humans). Therefore, the effect size is small in most cases.

Below, we re-created Figures 1 and 2 from the manuscript with a 10-day timescale of deuterium washout from body water (instead of a step function for the body deuterium concentration). The calculated slopes are, however, based on the simplified deuterium availability curve, i.e., the step function (hence identical to the ones in Figures 1 and 2 in the manuscript). The deuterium labelling curves are clearly delayed due to the slower turnover of deuterium in body water (Figures R1 and R2 below compared to Figures 1 and 2 in the manuscript), making the calculated initial label loss rate, $d^*(1+\epsilon)l_2$, in the POI a worse predictor of its labelling curve. However, the calculated initial, $p^*(0)$, and final, $p^*(1)$, label gain rates of the POI still provide a similarly faithful description of the true labelling curve. We, therefore, conclude that our results do not depend on the simplified body deuterium concentration curves. To avoid unnecessary complications, we prefer to show the simplified model in the manuscript, but have added a brief discussion on this in the revised manuscript (see line numbers 96-98 and 874-885).

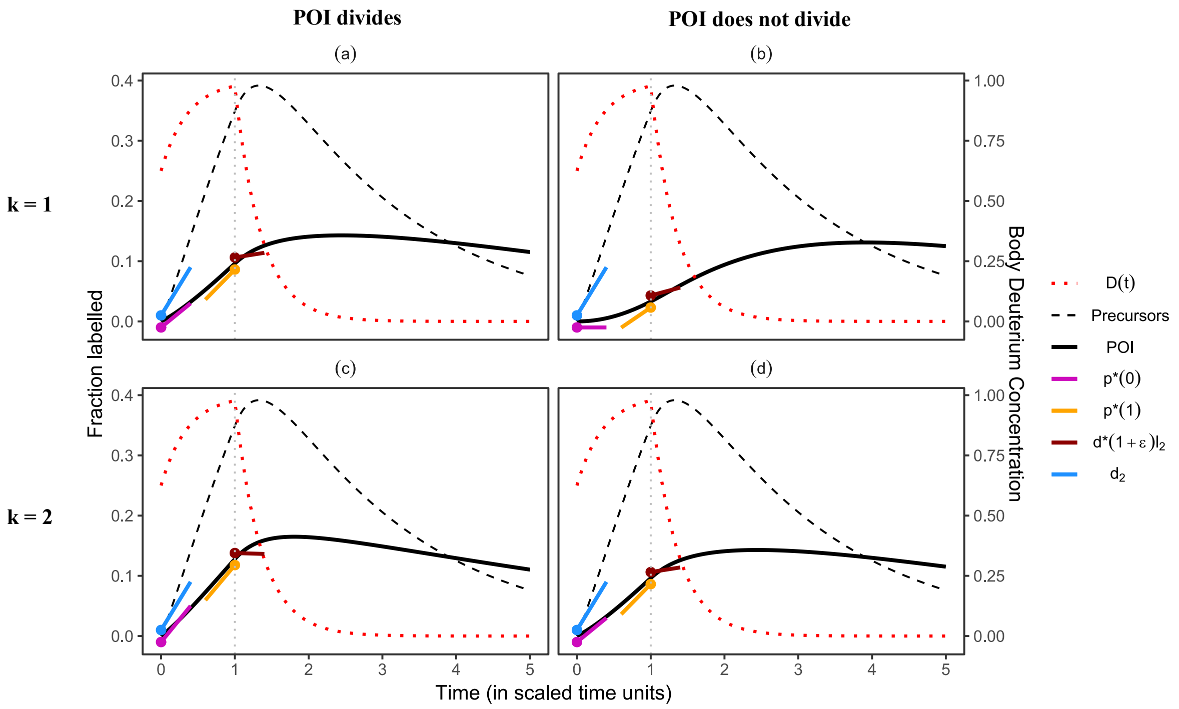

Figure R1: Calculated gain rates are a good approximation of the true labelling curves when precursors are faster than the POI. The turnover of deuterium in body water has a 10-day timescale in these simulations. See Figure 1 in the main text for other details. Note that the body deuterium concentration, $D(t)$, has been plotted on a different axis (on the right) to help comparison to Figure 1 in the main text.

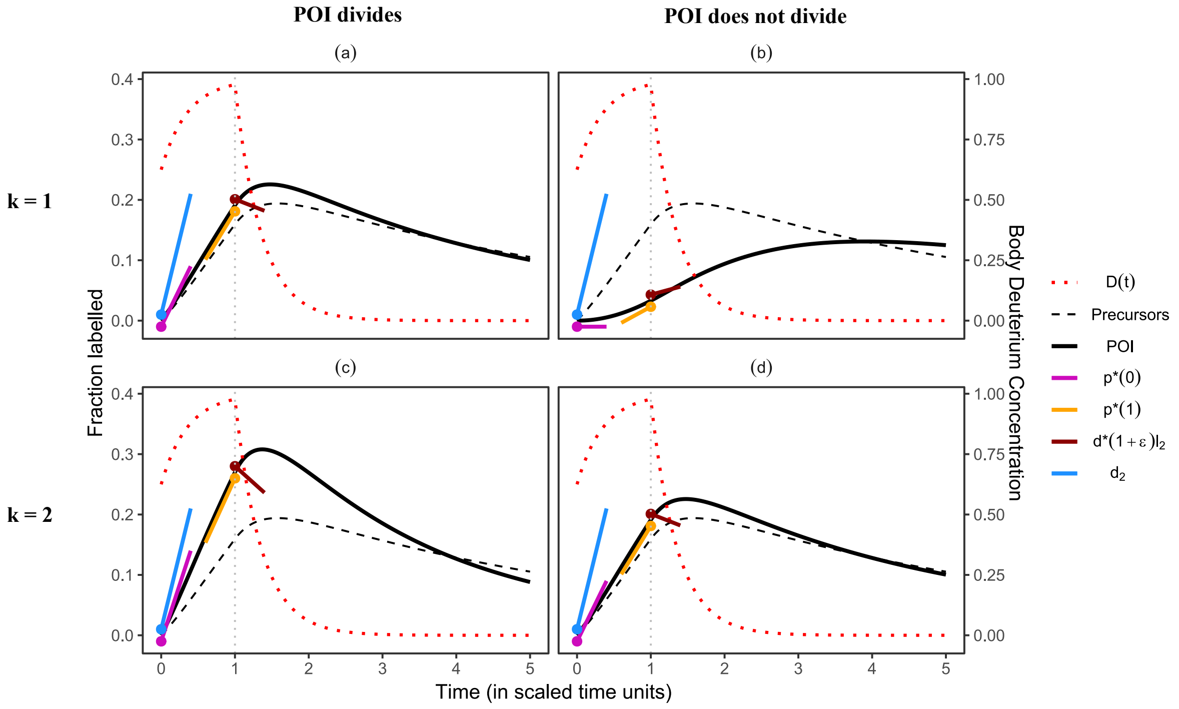

Figure R2: Calculated gain rates are a good approximation of the true labelling curves when precursors are slower than the POI. The turnover of deuterium in body water has a 10-day timescale in these simulations. See Figure 2 in the main text for other details. Note that

the body deuterium concentration, $D(t)$, has been plotted on a different axis (on the right) to help comparison to Figure 2 in the main text.

In response to the impact this simplification may have on discrimination between alternatives, below we show simulations of three populations in a chain under two different scenarios: deuterium availability modelled with a step function (Figure R3) and with a 10-day timescale (Figure R4). The first population is always an exponential-like, dividing population either followed by two non-dividing, exponential-like populations (Figures R3a and R4a) or followed by two non-dividing, waiting-box-like populations (Figures R3b and R4b). The second population (1.25-day timescale) in this chain has a slower turnover rate compared to the third population (1-day timescale). Like we reported in the manuscript, in the case with only exponential populations, the peaks of successive populations can only get lower (Figures R3a and R4a), whereas this is not necessarily the case for non-exponential populations (Figures R3b and R4b). Even though the difference between the peaks of the second and third non-exponential populations was much less pronounced when the turnover of body deuterium was slower (0.37 and 0.372 in Figure R4b compared to 0.28 and 0.29 in Figure R3b), the population with the slower turnover still had a lower peak, demonstrating that our claim holds even if the body water enrichment does not follow a step function.

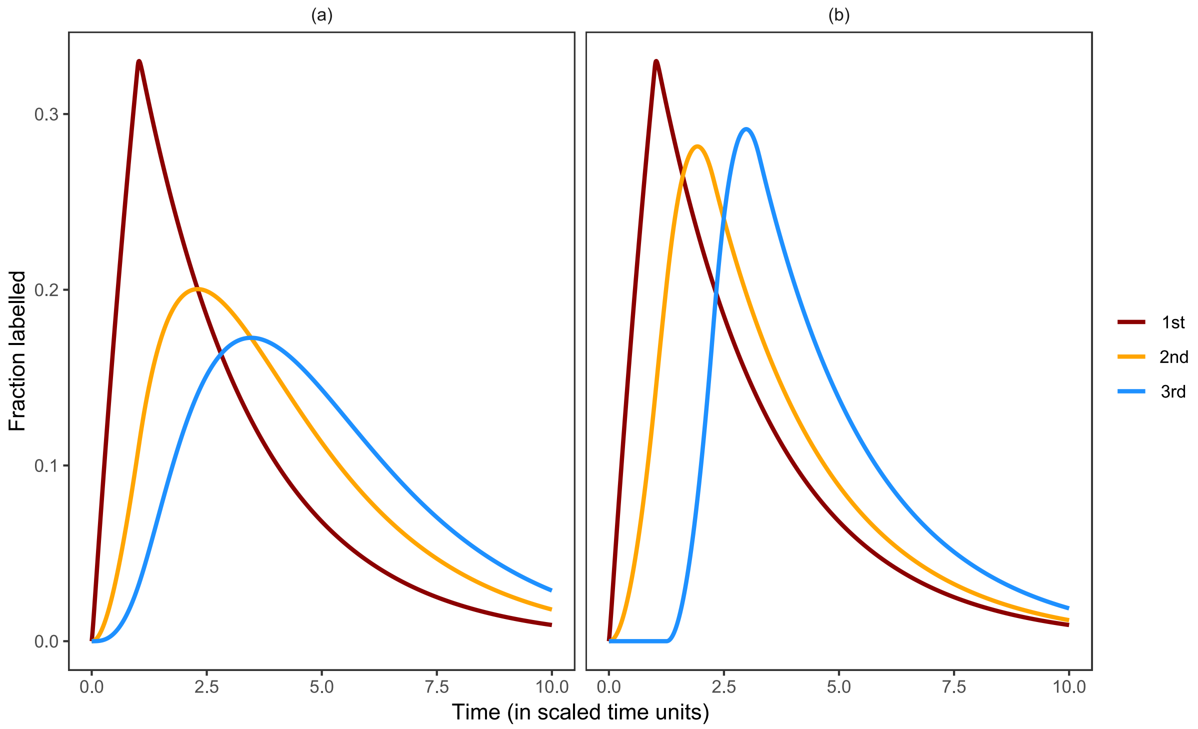

Figure R3: Exponential-like populations always have a labelling peak lower than that of their exponential-like precursor. The different lines represent the first, second and third population in the chain. Deuterium concentration in body water is described as a step function in these simulations (equation 1e in the main text).

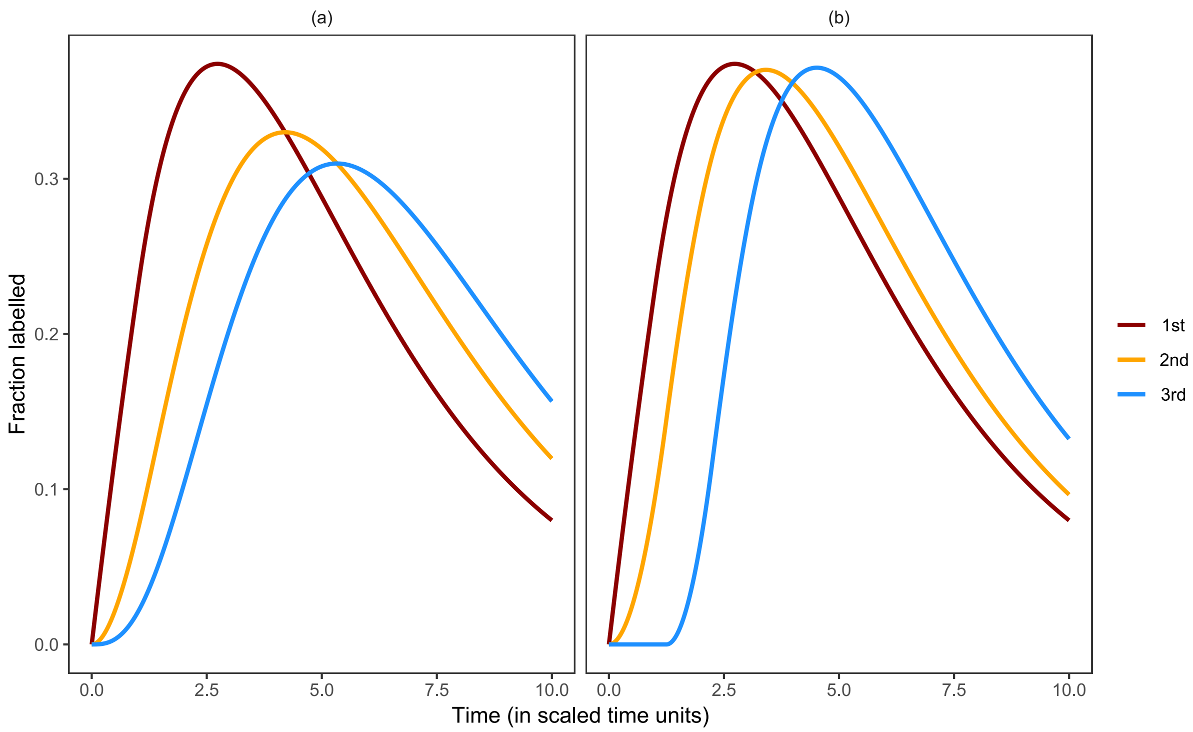

Figure R4: Exponential-like populations always have a labelling peak lower than that of their exponential-like precursor. The different lines represent the first, second and third population in the chain. The turnover of deuterium in body water has a 10-day timescale in these simulations.

Differentiation pathway. One of the main conclusions of the paper is that we need to know differentiation pathway of cells to the POI (i.e., labeling of precursors) in order to make proper inferences. I wonder if we ever know these pathways. Some recent papers challenge classical ways of hematopoietic cell differentiation (PMID: 24508463). So my question is how precisely do we need to know differentiation pathway? Or how many imprecisions in the knowledge of the pathway could there be so that turnover rate of POI is estimated with small or large errors? Some insights (discussion?) of this is needed if authors to make the conclusion they make.

We agree with the reviewer that these pathways may not always be (sufficiently) known. Even if we think we know these pathways, one can never exclude the possibility that progressive insights may prove previous models wrong. We, therefore, feel that our work makes two important points: i) the good news is that the inference of the POI's rates depends only on its immediate precursor (equation 2h in the manuscript), and that even the true turnover rates of that precursor are not required, as long as we know how much label is brought into the POI via the precursor, but ii) the bad news is that the lack of knowledge on the precursor population (e.g., which population it is, what its dynamics are, or how much label it brings into the POI) may introduce large errors in the estimates of the POI's rates (see Figures 3 and 4 in the manuscript). Our manuscript quantifies the potential errors of not considering a precursor population for several scenarios, and emphasizes the importance of knowledge on the immediate precursors and taking along their labelling levels when interpreting the POI's rates.

We have added these points in the Discussion section of the manuscript (see line numbers 886-893).

Comparing models to data. It is nice that authors compare models to data. However, both examples are not exciting for the main text. For memory CD8 T cells (Figure 5), there is no way that memory T cells have 5 days lifespan, so obviously the ES model is incorrect (assuming that all labeled cells come from the source/precursors). I think a better example here would be data on neutrophils and illustrating/repeating previous results showing how lifespan depends on the model would be more informative. (And it would be good for authors to comment on their Blood paper in Discussion). As for Ahmed et al 2020 paper data, I think a better analysis would be to assume different from the paper differentiation pathway and see if lifespan estimates depend on it. For example, there is a cell from which both 57+ and 57- cells are made at different rates -> how will this impact estimates of turnover rates? Authors may want to come up with other models that are not assumed in Ahmed et al. Panel B/C in Figure 6 are not useful as the model clearly does not fit the data (showing AIC would be useful).

With hindsight, we agree with the reviewer that the added value of our re-analysis of the memory CD8 T-cell data of Westera et al. (old Figure 5) was very limited and we have, therefore, removed this analysis in the revised manuscript. Our objective was to illustrate the large potential differences in turnover estimates if the correct labelling of the source is not considered. We do feel that the CD57 data by Ahmed et al. provide a beautiful example to illustrate this. We have, therefore, changed the analyses of these data in a way that strengthens this aim: we now show how the estimated rates of the POI change if the precursor is or is not taken into account. Even though the fits to the data look (almost) perfect in all cases, we see that the estimated turnover rates of the POI are quite drastically impacted when the precursors are ignored. We agree with the reviewer that it would also be very interesting to consider possible alternative differentiation schemes, but feel that this is beyond the scope of the current manuscript. We will consider exploring such alternatives in our future work.

Limitations. I did not see a part of discussion exclusively devoted to limitations of this work. Please address.

We have now added two paragraphs in the Discussion section, pointing out the limitations of this work and linked it to essential questions that should be addressed in future studies. We thank the reviewer for these discussion points.

Rewording novelty of the work. From my understanding of the literature, inability of simple models to discriminate between proliferation and source (the latter is precursors in the model) has been shown before (e.g., 9469816, 10799860, 12737664). Hence, focus of some previous work on "closed" populations (no source), e.g., memory T cells. In some ways, it is obvious since from labeling curves that give 2 slopes one can only estimate 2 parameters, and the model with 3 (source, division and death rates) will overfit such data. Also, the debate about neutrophil labeling clearly established that the model for source impacts estimates of cell lifespan. What authors do is to address this point in a more general framework, rather just for neutrophils.

That's fine but the abstract and text should be positioned from that perspective, by putting results in the proper context (and not overselling them).

Good point, thanks! In the revised manuscript, we have made it clear (in the Abstract and the Author Summary section) that we are building up on previous work that has pointed out that the dynamics of the precursors can impact the rates estimated for the POI. The novelty of our work lies in the fact that we go beyond the extremes (such as neutrophils, which have been addressed before), and mostly focus on understanding the interpretation of the estimated rates in the whole range of non-extreme cases.

Minor comments

I don't like commenting on nomenclature but I wonder if having name for POI as being separate, rather than N_2 or N (in the model with 1 population). This could also allow to have n precursor populations. But of course, this is just a suggestion.

Although we see value in this suggestion, for our model equations, we think our current nomenclature is the most effective and intuitive: the POI is always the final population (for example, the 3rd in a 3-population ES model) and the precursors are numbered accordingly. In response to the reviewer's point, we have renamed the different populations as "precursors" and "POI" in the figures of the revised manuscript.

I feel that most of derivations related to models could be moved to supplement, most results could be stated in the text with giving one formula results or focusing on the figures. Or perhaps moving main derivations into Materials and methods (currently missing from the paper).

As the derivations are not very lengthy, and because the readership of PLoS Computational Biology may like to follow the derivations, we prefer to keep them in the main text. However, we appreciate the reviewers concerns and have added two new section headings 'Mathematical Models' and 'Results'. We hope this will be sufficient to give the manuscript a familiar structure for readers.

When authors talk about kinetic heterogeneity model and just consider 2 populations with different turnover rates (eqn 3), I think authors are imprecise. This is a specific case of "explicit kinetic heterogeneity" model with n=2 populations, the full model in fact includes multiple sub-populations (infinite in the limiting case). Kinetic heterogeneity model should be referred to Asquith at el model with p and d* rates for increase and decline (12464572). Claiming that ES and KH models are identical is incorrect in general unless authors show this for KH model with variable (infinite?) number of sub-populations.

The referee is correct. We have not shown it for the general case (with n sub-populations). So, we have edited the manuscript to replace "kinetic heterogeneity" with "two sub-population kinetic heterogeneity", wherever appropriate.

I understand the desire to make estimates dimensionless but in some cases it is hard to connect results to other estimates we have for lymphocyte turnover. Would it be possible to have Toff time and then have all rates in per day units? This will make the paper less mathematical and better for biological audience -> this is PCB journal not BMB or JTB.

The reason why we strongly prefer to make our estimates dimensionless is that it allows us to compare gain and loss rates across varied experimental conditions, ranging from 6-hour labelling with deuterated glucose to 7-weeks labelling with deuterated water. We have provided an easy way to convert the rates reported in the manuscript into per day units. As we assume a labelling period of 28 days for our simulations, the rates can be either read as per month units or divided by 28 to convert them into per day units.

Figures should be revised to ensure that they can be interpreted by a color-blind individual and when printed on B&W printer (as I did).

We have revised the figures to make it appropriate for a color-blind reader and when printed on paper in B&W ink.

Captions for some figures lack details and titles are not very informative. For example, Figure 1 first sentence in bold should give the message of the figure. And also refer to relevant equations in the text. Table 2 caption is poor. Figures 3 and 4 should be squares with the same range for x and y, so the slope can be visually interpreted. Figure 5 caption is poor -> what are these data from previous paper? Are you suggesting I go and read that paper? I don't have the time. Explain what experiment was here. Also, I cannot see well panel letters a) b) c) for panels, make them bigger and bold. In b), the fit is not adequate (model does not fit some data) -> why/? Figure 6 should have SSR or (better) AIC to indicate the fit quality -> obviously in b/c model does not fit the data -> why do we can about these fits?

As per the reviewer's recommendation, we have improved the titles of the figures and the tables. We have also added more details about the figure or the table in its legend, and changed the axis wherever suggested. As discussed above, we have decided to remove the original Figure 5 in the revised manuscript. The original Figure 6, with the analysis of the CD57+ memory T cells, has now become Figure 5 and the analysis has been changed as described above. We have also reported the AICs of the fits in the caption of Table 3.

More detail should be given in how the models were fitted to data, i.e., was the labeling assumed to be step function? Is that a good assumption?

The referee is right in that we should have provided this information. Since cells were labelled over weeks with deuterated water in this experiment, we used the equation provided in the original article to describe the concentration of deuterium in body water. We have now added these details in the caption of the figure (see line number 775-778).

I wonder if knowing the lifespan/turnover of a cell population (e.g., memory T cells), one could make an estimate of the source when fitting s+p+d model to data. Perhaps this is something to be discussed.

We believe it would prove to be tricky to estimate the source when fitting the s+p+d model to data, even if the lifespan/turnover rate of a cell population would be known. The conclusion of our analyses is that knowing the dynamics of s helps to quantify d, because when s is known one knows the fraction of labelled DNA strands contributed by the source. One cannot conclude from our results, however, that knowing d will help to distinguish between s and p because both these processes (contribution from source and division) have the same effect on the labelling of the POI.

Line 702 - I don't think I ever have seen delay in labeling data, Figure 6 or 7 do not show it. So, while the model may predict such dynamics, it is not observed in typical experiments. Perhaps this is something to discuss.

We agree with the reviewer that real-world deuterium labelling data are hardly ever so granular that one can see such a delay. We have, therefore, removed this sentence from the manuscript.

723-724 - isn't that obvious? Seems that it is not a new result really and should be stated as such.

Although this point may seem obvious to the reviewer, most studies do not consider a partially labelled source population and, thus, interpret the estimated turnover rate as the true turnover rate of the POI. We have slightly changed the wording of the sentence in the revised manuscript.

Figure S6 - what is the usefulness of SSR here? Are you comparing alternative models?

The SSRs are indeed not very important in these cases, so we have removed them from the figures.

Reviewer #2: Swain, Borghans and de Boer have made a very nice contribution to the modeling literature illustrating the issues of trying to infer population turnover rates using cell labeling studies when there is also differentiation of cell types, particularly when slower proliferating cell types entering the pool of interest.

The major conclusion is that if the population one is investigating is not isolated (i.e. has rapid inflow), then estimates could be extremely skewed by rates in the precursor population (i.e. see d1 vs p* in Fig 3). This is a really well written and thought out article, and I just have a couple suggestions of potentially how to make it more broadly read, because I would love if others at least had an intuition of these concerns. Sincerely,

Daniel Reeves

We would like to thank the reviewer for his kind and supportive words for this piece of work.

First, it could be nice to provide a sort of executive level summary, ideally written for experimentalists, as to how worried/skeptical they should be about various results. I like your case studies and I think if you had a table/figure with some real numbers, for instance with CD4 or CD8 T cell proliferation/differentiation rates, you might be able to give a traffic signal type simplified (fine, ok, bad) message to describe what kinds of cell subsets are most likely to be mis-estimated, and which other ones are likely to be good enough.

We would like to thank the reviewer for this suggestion. We agree that an executive level summary might make this manuscript more attractive for experimentalists. We have added a box in the manuscript, along with a cartoon with a few example populations that detail the separate cases (line numbers 610-652). We chose not to provide real numbers as whether one should be worried also depends on other experimental conditions (for example, the length of the labelling period).

Another useful thing you might do is to explain precisely what extra measurements would really help in cases where misestimation is likely, for instance, you say: "Uniquely identifying the turnover rate of a POI, therefore, requires measurements (or knowledge) on the contribution of label from its precursors" -- does that require labeling and sorting? or are there other types of data that are a little easier to get that might be good enough to ensure estimates are reasonable (e.g. flow data https://journals.plos.org/ploscompbiol/article?id=10.1371/journal.pcbi.1005417)

That is an interesting line of thought and has been one of our primary questions: what additional information can help in accurately estimating all the parameters (source, division and loss) of a cell population? Unfortunately, we have not found any satisfactory answer yet. We have put forward our best approach at the moment, which is to sort and measure the labelling levels of the precursors of a POI as well. This extra information will help in estimating the turnover rate of the POI accurately. Alternatively, the fraction of Ki67+ cells in the precursors could be used as a measure of the precursor's dynamics.

Lastly, this was a comprehensive look at a particular nuance of labeling, though it left me wondering how two other factors, a) the dynamics of the free label, i.e. what if not a square pulse step function, and b) what about differentiation outward?, and/or what if inward and outward differentiation are balanced? If you do not want to explicitly model these considerations it would at least be useful to mention them in the discussion

The dynamics of the free label is an important point of discussion. We have added a discussion paragraph on this in the revised version of the manuscript (874-885). Also see Figures R1-R4 in response to Reviewer 1's question. In short, the approximations of the label gain rate of the POI are still valid under conditions of realistically slow dynamics of the free label (for example,

in deuterated water labelling experiments), whereas the initial label loss rate of the POI becomes more inaccurate.

In our analyses, we always considered populations to be at steady state. Thus, the outward and inward differentiation is always balanced. If the reviewer is referring to the outward differentiation of the precursors and inward differentiation into the POI, our model takes that into account with the term α, accounting for the scenario where the number of cells leaving the precursors might not be the same as those entering the POI. The turnover rate or the loss rate, d, takes both the process of death and outward differentiation (defined by the term α in equation 2b) into account. Interestingly, the term α drops out of the equation when the deuterated fraction in a population is calculated (equation 2h), showing that the fraction of a precursor that differentiates into the POI does not impact the deuterium labelling curve of the POI.

Minor:

-might be worthwhile to explain that deuterium is sometimes notated as in heavy water D2O

We have added these synonyms in the "Introduction" section.

-I was a little confused where intro ended and results began

Thank you. We have now separated the main text into a "Mathematical Models" and a "Results" section.

-in Fig1 and 2, I think a simple cartoon of the model could help me understand the vectors better, but I still don't quite understand how this works (especially backwards yellow)

We agree with the reviewer that the direction of the yellow vector was confusing. We have now re-done Figures 1, 2 and S1, and added more details explaining the coloured lines in the legends of the figures. The new figures should now also be readable when printed in B&W.

-Fig5b doesn't fit so well, do you have any explanation for this? Is there differentiation outward?

Unfortunately, the ES model could not explain the data any better than the previously used kinetic heterogeneity model. We do not have an explanation for this yet. With hindsight, we feel that this specific dataset may not be the best to illustrate the impact of not knowing the precursor on estimating the turnover rate of a POI. We have, therefore, removed this case study from the manuscript and now focus only on the CD57 data, which especially with our new analyses (as discussed in our response to Reviewer 1) showcases the issues that we are pointing out in the manuscript much more clearly.

-"Deuterium labelling is hailed as the current best technique to accurately measure the turnover rates of a cell population in vivo", probably needs a reference, or references simply to this being

a common tool, and again the same point of any insight you can provide to what other tools could help parse out situations where labelling struggles

We have added a reference to this statement. We agree with the reviewer that it would be very valuable to know what other tools/measurements can be used when labelling data is not available. We are currently exploring this question in a separate study. One of the tools could be to measure the level of Ki67 expression of the precursors as well as the POI, which should provide information about the dynamics of each population. We will explore and discuss this at length in our future works.

-very minor, appreciate you sharing all your code, though could definitely benefit from a bit more commenting if someone else is to use

That is a good point. We have improved the annotations in the final version of the codes.

-in this work (https://www.nature.com/articles/s41467-023-41521-1), we used D2O data measured in 5 CD4 subsets to estimate proliferation and differentiation rates of HIV infected cells, however, we relied on the original D20 rate estimates naively, reasoning that differentiation was slower than proliferation so ok to ignore, this might be another useful study to comment on, as I'd be very curious how much our results would have changed if we instead used your modeling approach.

We thank the reviewer for this suggestion. We agree that this provides another interesting dataset to test the ES model on, and would be happy to explore this in our future work.

-I wasn't able to see the companion paper, but sounds interesting

We aim to publish our work and the companion paper in the same issue of PLoS Computational Biology, such that the similarities and differences in the approaches and results of both papers can be most easily compared.

Reviewer #3: The manuscript by Swain et al. presents a comprehensive study aimed at exploring how the estimated turnover rate of the deuterium-labeled population of interest (POI) may be affected by the dynamics of its precursor population. The striking examples in Figures 1b and 2b show that the fraction of deuterium-labeled for the POI may increase even when no cell division occurs and when the deuterium is washed out. The manuscript is well-written, and I have only a few minor comments below.

We would like to thank the reviewer for his/her supportive comments, and provide detailed responses to the comments below.

Figure 6: Please report the estimated parameters for Panels (b) and (c), add the value for 'k' in the Figure 6 legend, and add equations for the 3 populations in Panel (a) to the supplement.

We have reported the parameters in Table 3 and added the value of k in the Figure legend. Instead of repeating the equations in the supplementary information, we have added references to the exact equations when describing the procedure in the main text (see line numbers 738-745).

In Figure 6, the turnover rate of the POI (CD57+CD4+ population) is estimated as d2=0.4/day, which would correspond to a lifespan of about 2 days for these cells. Does this value make sense? Some discussion of the immunological relevance of the estimate would be useful. It would be interesting to see how parameter d2 (and lifespan) depends on the value of 'k' when fitting the same dataset.

In this manuscript, we re-analysed CD57+ T-cell labelling data merely to illustrate how large the impact of ignoring the precursor population can be. We do not want to claim that these fits to the CD57+ T-cell data are the best description of the underlying biology. We, therefore, feel that discussing the biological impact or likeliness of the relatively short cellular lifespan estimates is beyond the scope of this manuscript. We do agree, however, that understanding the dynamics of CD57+ T cells is biologically relevant, and are, therefore, currently comparing alternative potential differentiation schemes to fit the labelling data of CD57+ and CD57- T cells.

Note, by the way, that the estimates in our paper are similar to those reported in the original publication, which provides the current best lifespan estimate for this population.

We have added some additional information about the impact of the value of k on the fits to the data and the resulting parameter estimates (see line numbers 787-797).

As it is impossible in many cases to distinguish the fitting between k=1 and k=2 (e.g., Figure 1a,d), it is not clear under which conditions there is a need to use k=2. Please explain.

The reviewer is correct in that in many cases, it may be nearly impossible to distinguish between the k=1 and k=2 cases, unless one is biologically informed. The case study of CD57+ memory T cells happens to be an example in which it *is* possible to distinguish between the k=1 and k=2 cases (see line numbers 787-797). In the manuscript, we suggested to always fit the more general k=1 case and then infer whether the same fit can be explained by the k=2 case as well. This will allow one to point out cases where k=1 describes the data better. However, without any additional information, one cannot claim that k=2 is the only possible explanation for a dataset.

Line 215: It is clear that Equation (8) does not follow from Equation 1c as stated in the manuscript "the labeling curve of a POI can be written as (from Equation 1c):", because Equation (8) is more general. Please correct the statement.

Thanks for pointing this out. We have changed the sentence into ‘generalizing equation 1c using time-varying parameters’.

Line 416: Should the reference to Swain et al. be added by mistake? It seems out of place.

This reference is indeed not necessary and does not have a DOI yet, so we have removed it.

There are multiple references to the paper by Yan et al., which is problematic since it appears to be under review and inaccessible to reviewers or readers. It might be better to make the current manuscript a standalone paper. Alternatively, based on the provided mention of Yan's manuscript, combining the current manuscript with Yan's paper would be beneficial for the reader, as Yan's paper extends the current framework to the higher value of k=2^i reflecting clonal expansion, which likely results in a relationship similar to the one between k=1 and k=2, only covering a smaller subset of fittings possible for k=1.

The reviewer is correct in that higher values of k likely cover smaller subsets of k=1 than the k=2 case.

The Yan et al. manuscript is an independent study with non-overlapping authors. Because the questions we are addressing in both papers are so related, we decided to submit both papers to PLoS Computational Biology at the same time, such that they can hopefully be published back-to-back in the same issue. We do think this would be very helpful for the reader. Importantly, the two papers are independent of each other’s analyses and are, therefore, truly standalone papers. Both papers clearly refer to each other’s work to highlight the links and differences between the two papers.
