# Supplementary material for: How are estimated cellular turnover rates influenced by the dynamics of a source population?
Source: PLoS Comput Biol. 2025 May 12;21(5):e1013052. doi: 10.1371/journal.pcbi.1013052 (PMC12129353; doi:10.1371/journal.pcbi.1013052)
Supplement: Attachment — Submitted filename: ChainRebuttal2.pdf [file pcbi.1013052.s004.pdf]

Dear Editor,

Thanks a lot for allowing us a second revision. Reviewer 3 has a few minor suggestions, Reviewer 2 is happy with the current version, but Reviewer 1 makes several critical points. Most of these are thoughtful and helpful, some seem to be due to a misunderstanding about the time-dependence of the source, and some are sceptical about the potential solutions we are offering for the major problem we describe (i.e., also consider the precursor population). We have added explanations in places where the referee may have misinterpreted our original text. In this letter we respond to all suggestions, while referring to the revisions in the text. While making the revision, we realized that the proof in equation 12 was incomplete and we have therefore revised it to provide a complete proof. We hope you feel these responses and revisions are satisfactory and may even enable you to make a direct positive decision on the publication of this work.

Our responses to the reviewers' comments are given in red.

Yours sincerely,

Arpit C. Swain, José A. M. Borghans, Rob J. de Boer

Reviewer #1: Comments

The reviewer mentions 'time-varying source' and 's=s(t)' several times in their comments. We would like to clarify this point upfront, as there seems to be a misunderstanding here. The source in all the models in our manuscript is constant (and not time-varying) as we only model systems that are in homeostasis. The only difference between the proposed ES model and the commonly used IS model is that we allow the amount of label in the precursors flowing into the POI to be determined by the precursors' own turnover rate. Previously, the precursors were **assumed** to be fully labelled (which is the same as saying the precursors are extremely fast, or the label in the precursors follows that of the body water). This is why we write in the abstract that "Current models typically assume that the labelling in the precursors of the population of interest (POI) is proportional to the deuterium enrichment in body water/glucose. This assumption is not always true and it is known that this can have a significant effect on the rates estimated from labelling experiments."

Knowing immediate precursors. I am convinced by your arguments that knowing dynamics of labeling of immediate precursors is important -> it makes sense if s=s(t) in IS model, it will be important. What I found misleading is many statements about that point but only a short paragraph in Discussion that we may not know immediate precursors. I think this has to be stated better, including in the abstract, that we actually may not know for 100% immediate precursors for ANY POI. I would challenge you to give an example where immediate precursor is known 100%. An issue also may be that someone publishes a paper stating that population X differentiates into population Y but then few years later this is shown to be wrong (with some intermediate population or even that X and Y are not really related). To me, there is no solution here except of when one analyzes labelling data, two extreme models should be tried -> fully closed population and fully sourced population (and perhaps with time-dependent source). True

turnover rate will be in between estimates provided by the two models. Perhaps that's a useful point in your Discussion.

We agree with the reviewer that in many cases, one cannot be 100% sure of the identity and dynamics of the immediate precursors of a POI. We primarily point out the danger of ignoring the rates of the precursor population on the estimates of the POI. We totally agree that not knowing the true precursors of a POI is a fundamental issue, and therefore stress the need to always study the precursor population. A few examples of well-known precursors that are most likely correct are: naive T cells formed from thymocytes, memory T cells formed from naive T cells, and neutrophils in the circulation formed from bone marrow neutrophils. For other populations, identifying the immediate precursor may indeed be difficult, and will often remain uncertain. Nevertheless, we suggest that, with the current knowledge, even verifying the (expected) labelling of potential precursors is better than just fitting the IS model that assumes fully-labelled precursors. We now address this in more detail in two paragraphs in the Discussion of the revised version of the manuscript. We would prefer not to discuss this in the Abstract to make sure the Abstract remains understandable on its own.

We respectfully disagree with the proposal to fit a fully closed and a fully sourced model, as the IS labelling equations for a homogeneous fully closed and fully sourced model at steady state are the same, i.e., $\frac{dl}{dt} = d(D(t) - l)$. This shows that these two extreme cases are not giving two extreme values of d. The same is true for heterogeneous models, where $\frac{dl_i}{dt} = d_i(D(t) - l_i)$. This confusion is perhaps due to the misinterpretation of our source, which is not time-dependent (see our upfront response at the top of page 1).

Comparing explicit kinetic heterogeneity vs. explicit source models. I found the description of this point (lines 154-178) poor. I think what you are trying to say is that if one assumes closed population (no source) -- as explicit kinetic heterogeneity model by Ganusov et al. assumed -- and then consider population with a source, the models will give identical predictions. Thus, by model fit one cannot tell that the population is "closed" or has "source". You should state that explicitly in the beginning. But to be critical here, it is no brainer and I think previous models showed similar point (some of the early De Boer's papers on BrdU). Another point here (as I just realized) that the only true difference between IS and ES models is that in IS model source rate sigma is constant and in ES model it is time-dependent and explicit. However, if I use splines (or similar) to describe time-dependent sigma in IS model (e.g., CD57- cells in the data you analyzed assuming pathway CD57- -> CD57+), then this model would be nearly equivalent to the ES model. Please discuss.

We are indeed aware that the explicit kinetic heterogeneity model cannot distinguish between source and proliferation. As stated above, a fully closed or a fully sourced IS model (note that the individual sub-populations in an explicit kinetic heterogeneity model are usually modelled as IS models) have the same labelling equations. The point we are making is that in one of its parameter regimes, the homogeneous ES model is a weighted sum of two exponentials, just like the explicit kinetic heterogeneity model for two sub-populations. Thus, if one finds a good fit with an explicit kinetic heterogeneity model for two sub-populations, (1) it would be wrong to conclude that the POI is kinetically heterogeneous, and (2) the estimated turnover rate(s) could be wrong (if the POI were to have a significant but slower precursor).

Again, the ES model does not have a time-dependent source (sigma). The source itself is constant; it is only the level of label coming from the source that may be time-dependent. See our response at the top. It is true that one can use splines to describe the labelling of the precursors, and then forget about the d_1 of the precursors, but that is exactly the point we are making in the CD57 section. Knowing the labelling of the slow precursors is sufficient to reliably estimate the turnover rate of the POI because $l_1(t)$ can just be replaced by the spline function. We now address this in lines 145-147 of the revised manuscript.

Populations with a source or no source. I think it is critical to discuss situations in which POI can be closed. To me, populations of Ag-specific lymphocytes, e.g., LCMV-specific CD8 T cells, would be closed population (no source) in the absence of reinfection. In this case, we don't need the ES model, explicit kinetic heterogeneity model (with no source) will work fine. Perhaps this is an exception, though.

Yes, that is important because in such (maybe rare) cases the explicit kinetic heterogeneity model would indeed suffice, and we agree with your example. This has been added in lines 195-199.

Peak of labeling. Analyses in supplement clearly show that peaks of labeling in precursors may not always decline (e.g., Fig R3b, R4b), and the data in Fig 5 show higher peak for CD57+ cells than CD57- cells contradicting your model prediction if the model CD57- -> CD57+ is assumed. Experimentally we also expect to see some noise in measurements, so whether peaks are lower (or higher) may be hard to determine in some cases. Also, I wonder if this result of lower peaks in POI as compared to precursors survives non-exponential division times of cells. Finally, I also think that results with slowing accumulating label (D20) shown in the response letter should be put in the supplement.

The model describing the CD57 data is not a contradiction because the CD57+ cells are dividing. The labelling in the POI will have a lower peak compared to their precursors, but only in non-dividing (and exponentially modelled) populations (Fig R3a and R4a in the previous rebuttal).

We agree that in many cases it might be hard to compare peaks because of the noise in the data, however in cases where this is feasible, it is useful to notice this right away as one can immediately discard a set of models that cannot explain the data. For example, we are currently working on bone marrow data where the peaks are convincingly non-declining in successive populations, which provides a helpful insight given these results.

Non-exponential division times, like the DDEs describing a Smith Martin model, indeed fall in the category where peaks need not decline (Figure R3b and R4b in the previous rebuttal). We have added a paragraph on this in the main text (lines 779-783) and a section in the Supplementary Information.

Following the reviewer's suggestion, we have added the figures with slow label accumulation (D2O) to the Supplementary Information (Figures S2-S4, lines 330-332).

Analysis of experimental data. I like the new point that estimates of turnover rates of CD57+ T cells depend on the model but the problem here is that to make interpretation one has to assume relationship between CD57+ and CD57- populations. Authors here assume CD57- -> CD57+ pathway but how do you know this is correct? I think that would be a better point of discussion -> not that one has to take into account dynamics of precursors but that how these populations are related and how the rate of conversion determines estimates of the turnover rates. Given that accumulation of CD57+ cells in humans occurs over years most likely these populations are independent on the timescale of labeling, so the model assuming "rapid" differentiation of CD57- to CD57+ may be incorrect (e.g., differentiation may occur once in 10 divisions of CD57- cells). Please address.

The reviewer is correct that the CD57- -> CD57+ pathway shown in Figure 6 is an assumption and changing this assumption could affect the rates estimated for CD57+ cells. However, this assumption was made in the paper that we used as a case study (and is in line with the current consensus in the field). Our only message in this figure is that the estimated turnover rate of a POI (here, CD57+ cells) changes drastically if one lifts the assumption that their precursors turn over rapidly. Our manuscript provides the "warning" that the estimated division and death rate of the CD57+ cells strongly depends on this assumption. Like we mentioned above, as we are not discussing which population is the true precursor of the CD57+ cells, we only re-analyzed the data of one of the volunteers to illustrate our main point with an example. We deliberately refrain from interpreting the results, as we feel this is beyond the scope of the current manuscript. We hope to publish a full re-analysis of the dynamics of CD57- and CD57+ cells in the near future, in which we plan to address the questions raised by the reviewer.

Also, regarding the data. Please provide intuitive explanation of how labeling that reached 3% in 50 days (and similar decline of 2% in 50 days) matches 7 day turnover rate you estimate (line 748). This does not make sense to me, turnover rate must be much slower. Perhaps your estimate of 7 days comes from the assumption that labeling lasts 1 day and not 50 days (this is the issue of scaling the models that I pointed out to and that perhaps popped up here as huge error). I would still recommend using T as the duration of labeling and not 1 because I wonder if others may make the same mistake.

We understand the confusion by the Reviewer, but this is not due to the scaling of the length of the labelling phase: time in Figure 6 is not scaled. We agree that it is not good to scale time when analyzing true data, but it does help to generalize our findings in the current manuscript. The slowness of the curve in Figure 6 stems from the slowness in the individual's deuterium enrichment in plasma (hence this was not a mistake). We added this explanation to the figure caption in the revised manuscript. Also, note that the data of the paper were not normalized to the body water labelling levels. Thus, the maximum of the curves in Figure 6 is fc, where f is the predicted plateau of the body water enrichment, and c is the amplification factor. We prefer to use these data as they were previously published, and therefore have not normalized them.

I did not see an explicit section on limitations of the paper, limitations appear to be scattered in Discussion. Please put limitations in the specific place/paragraph(s) with appropriate

heading/first sentence, e.g., "Our work has following limitations" (or similar) per rigorous science.

Although we would personally have preferred to list and acknowledge the limitations of our work (we are grateful to the reviewer for pointing that out in the earlier revision) in an engaging fashion, on the reviewer's insistence, we have added a paragraph (lines 954-966) that starts with ‘Our work has a few limitations:’.

Minor comments

Line 146 - I am not sure if eqn. 2h can be fitted to data without knowing k. Then eq. 2h has 3 parameters (p_2, d_2, and k) but we typically think that labeling curves provide estimates for 2, so the model will overfit the data unless k is fixed. Please address.

The referee is partly correct, and that is why we have focused on fixing k=1 or k=2 (note that the companion paper (doi:10.1371/journal.pcbi.1012704) allows k to vary). The value of k may indeed be unidentifiable since the k=2 solutions are equal to the k=1 solutions for other values of d1, p2 and d2. However, it is not completely true that labelling data typically provide information for just 2 parameters. Many data sets have been successfully fitted with a 2-dimensional explicit kinetic heterogeneity model, which also has 3 parameters: alpha, d1 and d2.

Lines 15-16 in abstract are confusing. I think the issue is that precursor dynamics have not been taken into account, e.g., source has been constant in previous models. That's the main point of your work, I think (and perhaps having "mechanistic" description of the source).

We believe the referee's confusion stems from the misinterpretation of a constant source. The source in our models is constant, just like in previous models. It's only the level of label in the source that is modelled differently in our approach, taking into account the dynamics of the precursor, rather than assuming that the level of label in the precursors is always proportional to the label level in body water. Please see our upfront response at the top.

Line 25 - I think you should add the point that one must know, for 100%, that immediate precursors for POI are actually immediate precursors.

We feel that claiming that one needs to know the precursors with a 100% certainty is way too extreme if the alternative is to ignore the rate at which precursors are becoming labelled. This manuscript demonstrates that estimates for the division and death rates of a POI can be very off if one ignores the labelling of the precursors. If we want to make scientific progress, we need to consider the precursors even if we are not 100% sure about who they are. Perhaps one should consider several possible precursors if that is the objective, but one should always consider the current best precursor candidate(s). At least the consensus that the labelling of precursors can be ignored should be changed. We address this in more detail in the Discussion of the revised manuscript.

Line 397 -- what if labeling D(t) is not a step function? Then implicit kinetic heterogeneity model could give a delay in accumulation of label in POI.

We think the reviewer is asking particularly about explaining the initial delay in Figures S2b and S3b with a slow D(t). The data in these figures are generated with a D(t) that is a step function, so it would not be fair to explain these data with a D(t) that is not a step function. If the data were to be generated with a D(t) that is not a step function, we would expect an even longer initial delay in the data, which would be difficult to fit with the same D(t) that was used to create the data. Additionally, upon the reviewer's previous request, we removed the sentences 'Note that both examples are from labelling experiments with deuterated water and the body water enrichment approaches its asymptote slowly. Therefore, even a population that is entirely self-renewing can have an initial delay in gaining label (Figure 6).' from the originally submitted article (the reviewer's comments on line 702 in the first rebuttal). The reviewer had correctly pointed out that even though this is an interesting point to note, it had very limited practical impact as we have never seen such an initial delay in a real-world deuterium labelling dataset because of the sparsity of the datasets.

All figures: please put parameters used for simulations inside each panel. It is hard to go between figure and the caption to see what parameter values are.

We have included the parameter values in the panels of the new figures.

In Fig 2 b and d I was surprised by the huge difference in shape of two curves. Is that just because k=1 in b and k=2 in d? Perhaps this is worth noting.

Thank you. That is a good point. We have added a couple of sentences (lines 467-469 and lines 393-396) to emphasize this.

Authors spent a lot of time calculating limits/slopes. These are ok but ultimately nobody should use slopes to estimate rates, we typically fit the whole curve to the data (because estimating slopes may be hard due to noise in the data). Perhaps this is important to mention.

We agree and did not mean to recommend to calculate just the slopes as an alternative to fitting the model. We also do not do that in our analyses. Calculating the slopes at different points helps us to infer the parameters that govern a certain part of the curve. It also allows us to draw heatmaps (as shown in Figure 5, previously called Cartoon) of the parameter domains where things are expected to go wrong. We added a couple of sentences to clarify this. See lines 917-920.

When simulating dynamics of the model (by generating "data"), what was the time step used? How many total datapoints per curve? This info should be provided better but is completely absent now.

Thank you. We have added this information now.

I would suggest making Fig 3 and Fig 4 panels squared because ranges on x and y axes are the same. Same point for the "cartoon".

Figures 3 and 4, and Figure 5 (previously called Cartoon) now show square panels.

For the cartoon, I am not sure I buy the division into cell types... Perhaps just have some "names" as rapidly dividing POI and small source, slowly dividing POI, or similar. I don't know if we know which areas correspond to which cells (and which species - humans? Mice? Rabbits?)

We have added details about the species of the annotated populations in the Box.

Table 3 should explicitly show average turnover rates of the cells for different models. Currently it does not. Also, put average rate estimates in Figure 5.

We have changed the font of the average turnover rates for each scenario to **bold** for easy comparison and added the estimates to Figure 6 (previously called Figure 5).

I liked the point that a model with k=2 should not describe the Ahmed et al. data. This could imply that k=1 or that the assumed differentiation pathway is wrong. I think showing model fits with k=2 could be useful -> I.e., how wrong the model predictions are.

Thanks, this is an excellent suggestion. We have added the model fits for the k=2 case in the Supplementary Information.

First sentence of Figure 5 caption does not match the point of the figure. For example, panel a does not have CD57- population.

Good point. We have omitted CD57- from that sentence as these are indeed not our main POI.

Reviewer #2: Comments

Thanks for the responses and the work on the danger zone box. I hope this article gets appreciated as I think this is a concept that is relatively intuitive yet mostly ignored and as you show that might lead to large errors.

We are glad that we addressed all of the reviewer's concerns and thank the reviewer for their earlier comments, which helped us improve the manuscript.

Reviewer #3: Comments

The manuscript has undergone substantial revision and is significantly improved. The much better fit to the Ahmed et al. data in the new Figure 5 and the added cartoon are particularly appreciated. The authors addressed my previous comments, and I have only a few minor points regarding the newly added pieces.

The authors replaced the previous ES model-based Figure 6 with the current Figure 5, where each proposed model includes a kinetically heterogeneous population consisting of two sub-populations. The motivation for this change is described as: "We do this to allow the highest degree of freedom to the model to explain the data." I found this explanation confusing, as earlier in the text they stated, "the two sub-population kinetic heterogeneity model [6,9] turns out to be a special case of the ES model."

If I understand correctly, the three-population model in previous Figure 6 is somewhat equivalent to the current two-population model in Figure 5. It may be worth including the case of the ES model with three populations from the previous Figure 6 for comparison, as an alternative model.

We thank the reviewer for pointing out that these sentences were confusing. We have removed the sentence 'We do this to allow the highest degree of freedom to the model to explain the data' as it was in fact a sentence that referred to the model that was used in the previous version (pd* model).

For clarity, I strongly suggest adding the corresponding systems of equations used to fit the data in the different panels of new Figure 5 (perhaps in the supplement?).

Thanks, we have added the equations in the Supplementary Information.

It appears that the added cartoon is based on a simulation. Please add more details on how it was generated and add the corresponding numbers to the axes.

The previous version of the Cartoon (now called Figure 5) was just an artistic illustration. We have now improved the Cartoon by generating heatmaps from the derived approximations ($p^*(1)$) provided in Table 2. We have added these details in the Box.

The following points refer to lines in the revised version with corrections:

- Should the reported values in lines 810 (31%) and 925 (33%) be consistent? Please verify.
- Please add references for lines 959 and 962.

We thank the reviewer for pointing out the inconsistency. We have corrected this now. We have also added references for the mentioned lines.
